# The impact of the COVID-19 pandemic on the Galapagos Islands' seafood system from consumers’ perspectives

**DOI:** 10.1038/s41598-024-52247-5

**Published:** 2024-01-19

**Authors:** Mauricio Castrejón, Jeremy Pittman, Cristina Miño, Jorge Ramírez-González, César Viteri, Nicolas Moity, Solange Andrade-Vera, Renato Caceres, Michael K. Tanner, Gabriela Rodríguez, María José Barragán-Paladines

**Affiliations:** 1 Grupo de Investigación en Biodiversidad, Medio Ambiente y Salud, Universidad de Las Américas, UDLAPark 2, Redondel del Ciclista s/n, Quito, Ecuador; 2 University of Waterloo, Faculty of the Environment, 200 University Ave. W., Waterloo, ON Canada; 3 Flanders Marine Institute, Jacobsenstraat 1, 8400 Oostende, Belgium; 4 Charles Darwin Research Station, Charles Darwin Foundation, Puerto Ayora, Galapagos, Ecuador; 5 Center for Earth System Research and Sustainability (CEN), University Hamburg, Hamburg, Germany

**Keywords:** Environmental impact, Ocean sciences

## Abstract

The COVID-19 pandemic's early stages severely impacted global fisheries, particularly areas heavily reliant on imported food and tourism like the Galapagos Islands, Ecuador. To contain the spread of the virus, a full lockdown was implemented. However, the collapse of the tourism industry precipitated the worst economic crisis in the history of this multiple-use marine protected area. This paper examines the impact of the pandemic's early stages on consumption patterns and seafood security in the Galapagos from consumers' perspective, drawing on online surveys conducted during the lockdown. Our findings revealed pre-existing seafood insecurity across the archipelago, further exacerbated by the pandemic on the least-populated island. Nevertheless, the seafood system displayed moderated resilience to the pandemic’s socioeconomic disruptions. A variety of adaptive responses were adopted by Galapagos residents to cope with the lockdown. Consumers modified their seafood consumption habits, while fishers adapted their harvesting and marketing strategies. Such adaptive responses were shaped by the unique socioeconomic characteristics of each inhabited island and the ability of seafood suppliers to shift from a tourism- and export-oriented to a resident- and domestic-oriented market. This transition has created novel opportunities to foster a systemic transformation of the Galapagos seafood system to enhance its resilience against future crises caused by new pandemics, climate change, or other natural and anthropogenic drivers of change.

## Introduction

The COVID-19 pandemic caused an unprecedented impact on the fishery sector worldwide^1,2^. In its early stages, the pandemic disrupted global seafood supply chains due to restrictions on the frequency of international and domestic flights, as well as drastic shifts in consumer demand caused by the unexpected closure of restaurants and markets as lockdowns were imposed^3^. Fishers and seafood consumers coped with and adapted in different ways to the economic perturbations caused by the pandemic according to their adaptive capacity, i.e., their capacity to anticipate and respond to change, minimize the consequences, recover, and take advantage of the new opportunities created^4,5^. In response to the COVID-19 pandemic, most consumers reduced their demand for fresh and high-value products, while the consumption of frozen, canned, marinated, and smoked seafood products increased globally^6^. At the same time, small-scale fishers adapted their distribution channels and marketing strategies, mainly through direct sales and delivery services to households^1^.

During the initial stages of the COVID-19 pandemic, lockdowns were an effective strategy to contain the virus's spread^7^, particularly in Small Island Developing States (SIDS)^8^. Despite these measures, SIDS were highly vulnerable to the pandemic due to their substantial reliance on imported food and tourism to uphold their populations’ livelihoods. This reliance made these complex social-ecological systems particularly susceptible to the pandemic’s detrimental impacts on food security and the economy^9^. For this reason, SIDS represents unique case studies to examine the resilience of food systems in geographically isolated regions amidst global socioeconomic disruptions. This knowledge it is instrumental to identify the vulnerabilities of food systems and to devise a variety of adaptation and transformation pathways to increase their resilience to diverse anthropogenic and natural drivers of change^10^.

Several studies have been conducted to assess the impact of the COVID-19 pandemic on SIDS' small-scale fisheries and food systems. Most of these publications evaluated case studies from Pacific Island countries, Papua New Guinea, Timor-Leste, and the Caribbean^8–10^. Conversely, few studies have looked at the impact of the COVID-19 pandemic on the inhabited oceanic islands situated in the Eastern Tropical Central Pacific (ETCP), which embraces the Exclusive Economic Zones of Costa Rica, Panama, Colombia, and Ecuador. While these islands are not formally classified as SIDS, they confronted similar challenges during the early stages of the COVID-19 pandemic.

The ETCP is one of the world's most important biodiversity hotspots, with large and small-scale fisheries contributing significantly to the economies and food security of not only Latin America, but also their main export markets, the United States and Europe^11,12^. The Eastern Tropical Central Pacific is also relevant because it embraces a unique network of fully protected and multiple-use marine protected areas (MPAs) listed by UNESCO as natural World Heritage sites, including Isla Coiba (Panama), Isla del Coco (Costa Rica), Isla Malpelo (Colombia), and the Galapagos Islands (Ecuador)^13^.

Global systematic literature reviews addressing the COVID-19 pandemic’s impact on Marine Protected Areas (MPAs) reveal a limited number of studies within the ETCP^14,15^. Such studies were conducted in the Galapagos Islands^16–18^, standing as one of the world's most iconic multiple-use MPAs. Even though this volcanic archipelago is not categorized as a SIDS by United Nations^19^, it meets the same criteria. Like SIDS, Galapagos is characterized by a small population, remoteness, vulnerability to external economic and environmental shocks, and high reliance on imported food and tourism to sustain its economy and food security^20,21^. These features made the Galapagos’ human population vulnerable to global crises, such as the financial crisis 2007–2009^22^ and the COVID-19 pandemic^18^.

Between March 16^th^ and July 1^st^, 2020, a full lockdown was implemented in the Galapagos Islands by the Ecuadorian government to protect the local human population from COVID-19, together with the prohibition of commercial flights to and from the archipelago. Both measures were effective in containing the spread of the virus during the early stages of the pandemic. However, the unexpected and drastic decline in the number of tourists, and the slow recovery of the tourism industry after the lockdown, impacted the economy and lifestyle of more than 33 000 residents^23^, causing the most severe economic crisis in the history of the Galapagos.

The COVID-19 pandemic forced management authorities to shift their focus away from the conservation of Galapagos protected areas toward healthcare, food security, and economic reactivation of the local population^16,18^. Despite this shift in management priorities, few studies have been conducted to determine how the COVID-19 pandemic affected the Galapagos seafood system. This knowledge is essential for identifying opportunities to transform the Galapagos' small-scale fisheries using science, technology, and innovation. This will contribute to enhance the resilience of the local seafood system against the social-ecological impacts provoked by new pandemics and other global drivers of change.

Viteri et al. (2022) assessed the socio-economic impacts of the COVID-19 pandemic on the Galapagos small-scale fishing sector and the adaptive responses used by Galapagos fishers to face the COVID-19 pandemic, providing policy recommendations to enhance the resilience of the Galapagos small-scale fishing sector. In contrast, this paper examines the pandemic's impact on the Galapagos’ seafood system from consumers' perspective, based on online surveys conducted during the lockdown. We define “seafood” as any edible aquatic species consumed by Galapagos residents, including fish, crustaceans, and mollusks. We evaluated the impact of the early stages of the pandemic on the Galapagos seafood security and consumption patterns. Based on FAO^24^, we define seafood security as the ability of people to have physical, social, and economic access, always, to sufficient, safe, and nutritious food that meets their dietary needs and food preferences for an active and healthy life. This concept includes as pillars the availability, affordability, accessibility, and utilization of seafood. We identified the adaptive responses used by seafood suppliers during the lockdown and discussed how these responses represent an opportunity to increase the resilience of the Galapagos seafood system to new pandemics, climate change, and other natural and anthropogenic drivers of change. In this context, resilience represents the capacity of seafood consumers and fishing communities to cope with, adapt to, and change to sustain the seafood system and human well-being within a desirable state^25,26^.

## Methods

### Study area

**Figure 1:**
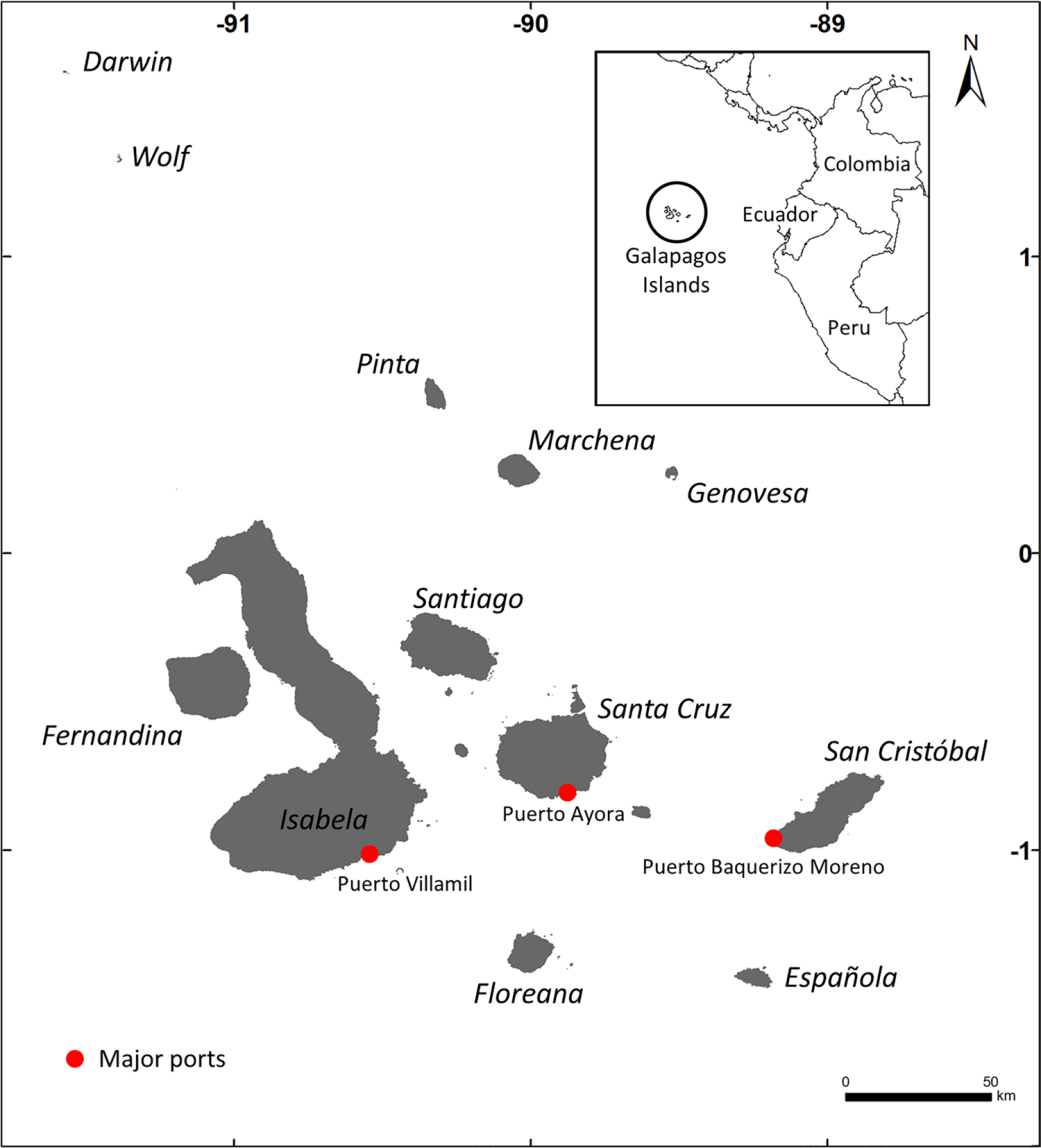
The Galapagos Islands, Ecuador with major port indicated by red dots.

The Galapagos Islands, situated 1200 km west of mainland Ecuador^27^ (Fig. 1), were inhabited by over 33,000 residents before the COVID-19 pandemic^23^, with 96% of the land designated as a national park^27^. The population is a mix of native residents (36%) and immigrants (63%), from other provinces of the Ecuador (Guayas, Tungurahua, and Manabi), with a small percentage (1%) born abroad^28^. The main inhabited islands, San Cristobal, Santa Cruz, and Isabela (Fig. 1), each have unique geographic and socioeconomic features, affecting their population density, fishing activities, and land-based tourism infrastructure (Table S1). San Cristobal has a moderate population, and a developing tourism infrastructure. It also hosts the highest number of full-time and part-time fishers (174). Conversely, Santa Cruz, although having fewer fishers (136), it stands out for being the most populated island with the highest number of hotels and restaurants. Finally, Isabela has the smallest population, the lowest number of fishers (100), and the fewest land-based tourism facilities (Table S1).

Approximately, 80% of the Galapagos economy depends on tourism, which employs approximately 60% of the residents^20^. The economic boom of tourism has driven steady demand for infrastructure, goods, and services in the archipelago, making the demand for food, water, and energy unsustainable^29^. As locally produced food is not sufficient to meet the demand of residents and tourists, the food security of the Galapagos is highly dependent on food imports from mainland Ecuador. These imports consist primarily of processed and ultra-processed foods, which usually are cheaper and more readily available than locally sourced fresh produce^30^. The dependence of Galapagos residents on industrialized food imports caused by high prices and inconsistent availability and quality of fresh produce, coupled with sedentary lifestyles, has resulted in unhealthy dietary habits^31^. Consequently, the Galapagos Province reports the highest prevalence of obesity and overweight conditions in Ecuador^32^.

In March 1998, the Galapagos Islands and its surrounding open waters were designated as a multiple-use marine protected area of 141,100 km^2^^27^, known as the Galapagos Marine Reserve (GMR). Since then, only small-scale commercial and subsistence fishing by Galapagos residents has been authorized inside the reserve^33^. Before the pandemic, there were 1100 fishing license holders and 333 vessels registered by the Galapagos National Park Directorate (GNPD). However, only 36% and 44% of fishers and vessels registered remained active in the fishing activity as full-time or part-time fishers^34^. A fishing license is required to participate in small-scale commercial fishing. It grants the holder the right to fish any type of shellfish and finfish species commercially permitted in the reserve. Galapagos residents can engage in subsistence fishing without a fishing license. However, a special license issued by the GNPD is required, restricting this activity to areas close to populated centers. During the lockdown fishers were organized in five fishing cooperatives: COPROPAG and COOPPABAPE in Puerto Ayora (Santa Cruz), COPESAN and COPESPROMAR in Baquerizo Moreno (San Cristobal), and COPAHISA in Villamil (Isabela)^35–39^.

Approximately, 68 marine species are commercially exploited in Galapagos, being the most consumed yellowfin tuna (*Thunnus albacares*), wahoo (*Acanthocybium solandri*), sailfin grouper (*Mycteroperca olfax*), and mottled scorpionfish (*Pontinus clemensi*)^35–37^. Galapagos’ food security is also reliant on frozen and processed seafood imported from mainland Ecuador, mainly shrimp (*Penaeus* sp.). However, before the pandemic, other imported species have been reported, including octopus (*Octopus* sp.), squid (Loligo sp.), snake eel (*Ophichthus* sp.), Nile tilapia (*Oreochromis niloticus*), Atlantic salmon (*Salmo salar*), corvina (*Cynoscion* sp.) and South Pacific hake (*Merluccius gayi*)^35^. Frozen and processed tuna is also imported to meet the local demand for canned seafood, despite the availability of fresh tuna in Galapagos. The main seafood purchasing venues are docks, seafood stores, fishing cooperatives, municipal markets and fairs. Seafood consumption per capita in Galapagos (10.6 kg) is higher than mainland Ecuador (7.7 kg), but lower than the average in Small Island Developing States (12.6 kg)^38,39^.

Before the pandemic, the annual fish demand in the Galapagos was approximately 871 t, with residents consuming 31% (272 t) and tourists consuming the remaining 69% (599.5 t)^40^. Tourists aboard cruise ships consumed up to 197 t of fish and 51 t of shellfish annually^35^. Moreover, the islands shipped about 30% of tuna landings (58 t) to mainland Ecuador, retaining 70% (138.5 t) predominantly for tourist consumption^40^. However, the COVID-19 pandemic brought about drastic changes. The number of visitors dropped 73% from 271,238 in 2019 to 73,000 in 2020^41^. This downturn forced many residents to migrate to mainland Ecuador in search of employment, reducing Galapagos population to 28,523 by December 2022^42^. The sharp decline in tourists and residents, coupled with closures of restaurants, hotels, and cruises, severely impacted seafood demand. Due to the collapse of the tourism industry, Galapagos experienced a market saturation of fresh and frozen fish, causing a substantial surplus^18^, particularly in the most populated islands. Market saturation was exacerbated by the fishing cooperatives’ inability to transport fish landings to mainland Ecuador due to the disruption of commercial flights. Consequently, seafood prices decreased, negatively affecting the economy of the small-scale fishing sector^18^.

Transportation, a crucial component of Galapagos' supply chain—responsible for 80% of its total food supply^43^—was hampered due to lockdown restrictions, reducing the supply of essential goods by 50–60%^18^. Some residents faced these challenges by embracing a barter-based economy, exchanging goods and services for essential supplies. Additionally, governmental and citizen initiatives emerged to assist those families facing financial hardships, including the distribution of basic food kits and fish donations from fishers^18^.

### Study design and sampling

During the lockdown, the authors resided in Puerto Ayora, Santa Cruz Island, Galapagos. Detailed observations were conducted from March 17th to April 17th, 2020 on the adaptation of seafood marketing practices, adjustments in distribution channels, and shifts in consumption patterns during the early stages of the pandemic. Insights gathered from these observations were used for the creation of an online survey, comprising 28 close-ended questions hosted on Qualtrics (refer to Supplementary Information for survey details). The survey gathered comprehensive data on seafood consumption habits during the lockdown. It analyzed aspects such as preferred species and products, purchasing frequency, consumption volume, and chosen shopping venues. It also explored changes in marketing and distribution channels, and evaluated seafood availability, affordability, and quality, considering various demographic factors like region of origin, island of residence, gender, age, migratory status, education level, individual monthly income, and economic sector. The collected data were used to analyze the impact of the early stages of the COVID-19 pandemic on seafood security and consumption habits. They also facilitated the identification of adaptive strategies employed by seafood consumers and seafood suppliers to cope with the socioeconomic perturbations provoked by this global driver of change. For widespread reach and representative participation, the survey was disseminated across various digital platforms, including Facebook, Twitter, WhatsApp, and email. It targeted individuals aged 18 years and over who were residents of the Galapagos Province before and during the lockdown. The survey was launched six weeks after the beginning of the lockdown and remained active for 35 days, from April 28th to June 2nd, 2020.

Participants were questioned about the frequency and quantity of seafood (fresh/frozen and canned) consumed before and during the lockdown, utilizing predefined categories (Table S2). These ordinal variables were then transformed into numeric ones to quantify the effect of the lockdown and demographic variables on seafood consumption habits (Table S2). Additionally, participants were also asked to describe the most consumed aquatic species before and during the lockdown through multiple-choice questions. To understand the lockdown’s impact on seafood consumption habits, the survey included questions about participants' pre-pandemic dining practices and the frequency with which they dined out during a typical week. Multiple-choice questions were employed to investigate consumers' adaptive responses to the closure of restaurants, including shifts in seafood purchasing communication methods, and marketing strategies. Likert scale questions were used to assess lockdown’s effect on various aspects of seafood security in Galapagos, including availability, quality, and affordability, with paired questions allowing for a comparison between pre-lockdown and lockdown responses. Additionally, the survey explored the potential persistence of altered consumption patterns and adaptive strategies post-lockdown, asking participants whether they intended to keep the newly adopted seafood consumption rates, supplier preferences, and communication methods once the lockdown ended.

Given that the primarily aims of this study is to examine seafood consumption patterns from consumers perspectives, we made a strategic decision to exclude fishers from the survey to avoid biases and divergent perspectives from the production side of the seafood industry. By focusing our research on consumers, we aim to gain a clearer understanding of demand-side behaviors and preferences of Galapagos residents. While this methodological approach was carefully chosen to align with our study's goals, we acknowledge that incorporating the perspectives of fishers in future research is crucial. Doing so would significantly enhance our understanding of the Galapagos seafood system, providing a holistic view that encompasses both the demand and supply aspects of the Galapagos seafood system.

### Data analysis techniques

Frequencies and percentages were calculated for the variables described in Table 1. The results were displayed using bar charts and Likert scale plots. In some cases, instead of displaying variations in participants' responses before and during the lockdown, we calculated the percentage of change to facilitate the comprehension of the shifts in seafood consumption habits that occurred between both periods. We conducted a discriminated data analysis per island (Santa Cruz, San Cristobal, and Isabela) to assess geographic differences in seafood consumption patterns and adaptive responses.

**Table 1:** Variables utilized to describe residents’ seafood consumption habits before and during the lockdown implemented in the Galapagos Islands, Ecuador from March 17th to July 1st, 2020.

Variable	Definition	Categories/levels
Seafood consumption frequency,	Number of times seafood is consumed within a specified period	Never consume seafood; once per month; once every 15 days; once per week; twice per week; three days per week; more than four days per week; daily
Average weekly seafood consumption	Average amount of seafood consumed per week measured in pounds	Half a pound or less; one pound; one pound and a half; two to three pounds; more than three pounds
Seafood type	Different kinds of seafood consumed	Fresh/frozen, canned
Period	Interval at which seafood consumption was observed	Before lockdown (pre-lockdown), during lockdown (lockdown)
Weekly frequency of out-of-home seafood consumption	Number of times seafood was consumed outside home per week, before the lockdown	Never, sometimes, often, usually, always
Adaptive responses	Adjustments or changes in seafood consumption habits in response to the lockdown	Canned seafood consumption; consumption of other types of food; cooked seafood home delivery (entrepreneurs); cooked seafood home delivery (restaurants); raw seafood home delivery; other
Aquatic species consumed	Fish and shellfish species that are consumed before and during the lockdown	Chiton; grape eye; mullet; octopus; sailfin grouper; scorpionfish; shrimp; slipper lobster; snapper; spiny lobster; swordfish; wahoo; white-spotted sandbass; yellowfin tuna; other
Seafood shopping venues	Places where seafood was purchased before and during the lockdown	Dock; fishing co-op; municipal market and fair; raw seafood vending vehicles; seafood home delivery (stores); seafood home delivery (co-op); seafood home delivery (fisher); seafood store; supermarket; other
Seafood purchasing factors	Elements that influenced the decision to buy seafood before and during the lockdown	Avoiding leaving home; closeness to home; customer support; out of habit; price; quality; sanitation; trust; others
Communication methods	Seafood information sourcing methods before and during the lockdown	App; cellphone call; Facebook; family and friends; seafood supplier; text messages; webpage; WhatsApp; none; other
Seafood availability	Ease of access of seafood in the local market before and during the lockdown	Never; sometimes; always
Seafood quality	Freshness, taste, and overall condition of seafood before and during the lockdown	Never; sometimes; always
Seafood affordability	Economic feasibility of buying seafood, considering its price relative to consumer income or willingness to pay, before and during the lockdown	Never; sometimes; always
Purchasing seafood post-pandemic intention	Willingness to maintain the same seafood purchasing habits adopted during the lockdown	No; maybe; yes
Seafood consumption post-pandemic intention	Willingness to maintain the same seafood consumption rates adopted during the lockdown	No; maybe; yes
Post-lockdown seafood procurement communication continuity	Willingness to continue using the same communication channels for obtaining seafood as were used during the lockdown	No; maybe; yes

To evaluate the effect of lockdown and geographic, demographic, and socioeconomic explanatory variables on the frequency and amount of fresh/frozen and canned seafood consumption before and during the lockdown, we built boosted regression trees (BRT). The BRT is a flexible non-parametric statistical and machine learning technique for classification and regression^44,45^. BRT models prioritize predictive accuracy and the elucidation of relationships within data over conventional measures of statistical significance, such as p-values. Consequently, the BRT models do not offer a formal significance test. Instead, their utility lies in their capacity to handle different types of predictor variables and their ability to model complex nonlinear relationships, enhancing the understanding of the patterns within the data^45^.

We built two BRT models. The first one predicted seafood consumption frequency, based on the following independent demographic and socioeconomic variables or predictors: gender, age, region of origin, island of residence, migratory status, individual monthly income, and economic sector. We included the type of seafood consumed (fresh/frozen and canned) as an independent variable to distinguish how seafood consumption rates could be influenced by the types of seafood products available in each inhabited island, before and during the lockdown. In the survey, respondents indicated consumption of either fresh/frozen or canned seafood, or both. This approach acknowledges that an individual may consume different types of seafood, with the frequency of consumption for each type potentially varying. We evaluated whether shifts toward or away from particular types of seafood products are discernible amidst the changing socio-economic conditions provoked by the lockdown, and how these shifts might be related to other demographic and socio-economic factors. The second BRT model focused on predicting the average weekly seafood consumption, utilizing the same set of demographic and socioeconomic variables, with the exclusion of “Seafood type”. We excluded this variable because fresh and frozen seafood consumption is quantified in pounds, whereas canned seafood is usually measured in grams. This discrepancy in measurement units made it inadequate to directly compare the data for fresh/frozen and canned seafood within the same model. This adjustment simplified the model and ensured that the analysis was methodologically sound. Consequently, the analysis evaluated exclusively the impact of demographic and socioeconomic factors on the average weekly seafood consumption of seafood in pounds.

Individual terms in BRT models are simple trees, created by recursive binary splits constructed from predictor variables and combined to optimize predictive performance, which are fitted in a forward, stagewise fashion^44^. BRT models can handle missing values in continuous and categorical predictors, as well as outliers, variable interactions, collinear variables, and nonlinear relationships between predictor and response variables^45,46^. Three parameters are required to fit BRT models^44^: (1) Learning rate (lr), ranging between 0 and 1, represents the rate at which the model converges on a solution; (2) Tree complexity (tc) refers to the ability of model interactions, represented by the number of nodes in a tree; and (3) Optimal number of trees (nt) required to increase performance prediction, which it is estimated based on the lr and tc. To enhance accuracy and reduce overfitting, we added stochasticity to the BRT model via a "bag fraction," which represents the proportion of data chosen at each step to build the model^44^. The bag fraction is expressed as a decimal between 0 and 1. We fitted BRT models with different combinations of tree complexity, learning rate, and bag fraction values. Ten-fold cross-validation of training data was used for each parameter combination to determine the optimal number of trees required to minimize deviance and maximize predictive performance. Then, during cross-validation, an independent testing dataset was used to predict response variables. The deviance explained and Pearson's correlation coefficient (r) were used to evaluate the predictive performance of each BRT model. The best predictive performance was achieved by a BRT model with a tree complexity of eight, a learning rate of 0.001, and a bag fraction of 0.6.

The Variable importance (VI) score was used to measure the relative influence of predictor variables on response variables. The VI scores are calculated by averaging the number of times a variable is split and the squared improvement that results from these splits^47,48^. The sum of VI scores is scaled so that it adds to 100, with higher numbers indicating a stronger influence on the response variable. We added a random number (RN) as a predictor to identify the most relevant predictor variables for modeling a response, following Soykan et al.^46^. Thus, the variables with higher VI scores than the RN were the most relevant in predicting seafood consumption frequency and amount in the Galapagos Islands. Then, we build partial dependence plots to show the effect of each predictor on response variables after accounting for the average effects of all other predictor variables in the BRT model, including the RN. The BRT model was fitted using the R statistical programming language, version 4.2.1 (R Development Core Team 2014). We used the “gbm” and “dismo” packages complemented with the brt.function code developed by Elith et al.^44^. ChatGPT-4 was used to improve the readability and language of the manuscript.

### Ethics

This research has been approved by the Human Research Ethics Board at the University of Waterloo, under permit ORE # 41749. All methods were performed in accordance with the Tri-Council Policy Statement: Ethical Conduct for Research Involving Humans (TCPS, 2nd edition). Informed consent was obtained from all participants. The authors declare that the research was conducted in the absence of any commercial or financial relationships that could be construed as a potential conflict of interest.

## Results

### Demographic profile of participants

Qualtrics recorded 372 participants. After excluding non-consenters, incomplete responses, respondents not residing in Galapagos before and during the lockdown, and those identifying exclusively as "fishers", a total of 225 valid responses remained for analysis (Table 2). Fishers were excluded to evaluate the perceptions of seafood consumers, rather than suppliers. Further exclusions were made for participants residing on Floreana Island, those possessing basic or no education, and individuals employed in the transportation sector. These four categories, related to the Island of residence, Education, and Economic sector variables, were each omitted as they were solely represented by a single participant. These adjustments allowed for a margin of error of 6% at a 95% confidence level, considering an economically active population consisting of 21,637 individuals^23^.

**Table 2:** Comparison between the demographic and socioeconomic characteristics of the participants of this study during the lockdown implemented in the Galapagos Islands, Ecuador from March 17th to July 1st, 2020, and the broader population of the Galapagos, according to the latest national population census^49^.

Factor	Variable	N = 225	%	N = 28,583	%
Gender	Female	121	57	14,203	49.7
	Male	91	43	14,380	50.3
	Unanswered	13			
Age group	18–25	10	5	Different categorization	
	26–35	65	31		
	36–45	82	38		
	46–60	47	22		
	> 60	9	4		
	Unanswered	12			
Region of origin*	Galapagos	74	35	9125	36.1
	Highlands	66	31	15,789	62.9
	Coast	55	26		
	Amazon	3	1		
	Foreigner	15	7	330	1.3
	Unanswered	12			
Island of residence	Santa Cruz	153	68	17,233	60.3
	San Cristóbal	51	23	8300	29
	Isabela	21	9	3050	10.7
Migratory status	Permanent resident	185	83	Data not collected	
	Temporary resident	38	17		
	Unanswered	2			
Education	Secondary school	60	27	Different categorization	
	Undergraduate	115	51		
	Postgraduate	50	22		
Monthly income (US$)	No income	60	31	Data not collected	
	< 500	22	11		
	501–1500	58	29		
	1501–2500	34	17		
	2501–3500	16	8		
	≥ 3501	7	4		
	Unanswered	28			
Economic sector	Jobless	30	13	Data not collected	
	Tourism	62	28		
	Public service	41	18		
	Commerce	18	8		
	NGO	16	7		
	Academy	7	3		
	Multisectoral	25	11		
	Other	26	12		
Household dependents	None	30	13	Data not collected	
	One person	21	9		
	Two persons	70	31		
	3–4 persons	69	31		
	> 4 persons	34	15		
	Unanswered	1			

*Data from the 2015 Galapagos population census^28^.

Most participants were females, permanent residents, with one-third being native to Galapagos (Table 2). Most participants resided in Santa Cruz and San Cristobal Islands. Approximately, 69% of participants ranged between 26 and 45 years old, and over 70% hold at least a bachelor’s degree. Participants were mainly involved in tourism, public sector, commerce, and non-governmental organizations (NGO). About 13% were unemployed, while 28% had no income at the time of the survey. Most participants (46%) had an income between US$501 and US$2500 monthly (Table 2).

To assess the representativeness of our sample relative to the broader Galapagos population, we incorporated in Table 2 a comparison with data from the most recent national population census conducted between October and December 2022^49^. However, this comparison faces limitations due to the nature of the Galapagos population census. Several variables critical to our study were either not collected during the census or were categorized differently (Table 2). This disparity restricts our ability to perform a comprehensive comparison across all variables. Despite these challenges, it is noteworthy that our sample closely mirrors the census data in terms of the “Island of residence” variable. Additionally, for the “Region of origin” variable, we utilized data from the 2015 Galapagos population census^28^, finding a good alignment with our sample. The primary deviation observed was in the gender proportion, where our sample diverged from the national census figures (Table 2). As our survey did not favor one gender over another, such discrepancy could be attributed to inherent differences in the willingness to participate in surveys between genders.

### Seafood consumption patterns and adaptive responses during the lockdown

Before the pandemic, varied fresh/frozen seafood consumption patterns were observed across the inhabited islands (Fig. S1). Isabela showed diverse seafood consumption rates, which shifted towards a preference for weekly consumption during the lockdown. In San Cristóbal, daily seafood consumption increased during the lockdown, while Santa Cruz maintained a consistent bi-weekly consumption pattern (Fig. S1). Canned seafood consumption in Isabela increased during the lockdown, while Santa Cruz remained consistently low (Fig. S1). Before lockdown, the average weekly seafood consumption both Isabela and San Cristóbal was higher than Santa Cruz, with 42% and 34.5% of residents, respectively, consuming over three pounds per week (Fig. S2). In contrast, Santa Cruz exhibited a more evenly distributed pattern of seafood consumption. During the lockdown, a general trend towards moderated seafood consumption emerged in Isabela and Santa Cruz, while San Cristobal showed a preference for larger amounts of seafood, evidenced by a rise from 34.5 to 41% in the proportion of residents consuming over three pounds per week (Fig. S2).

**Figure 2:**
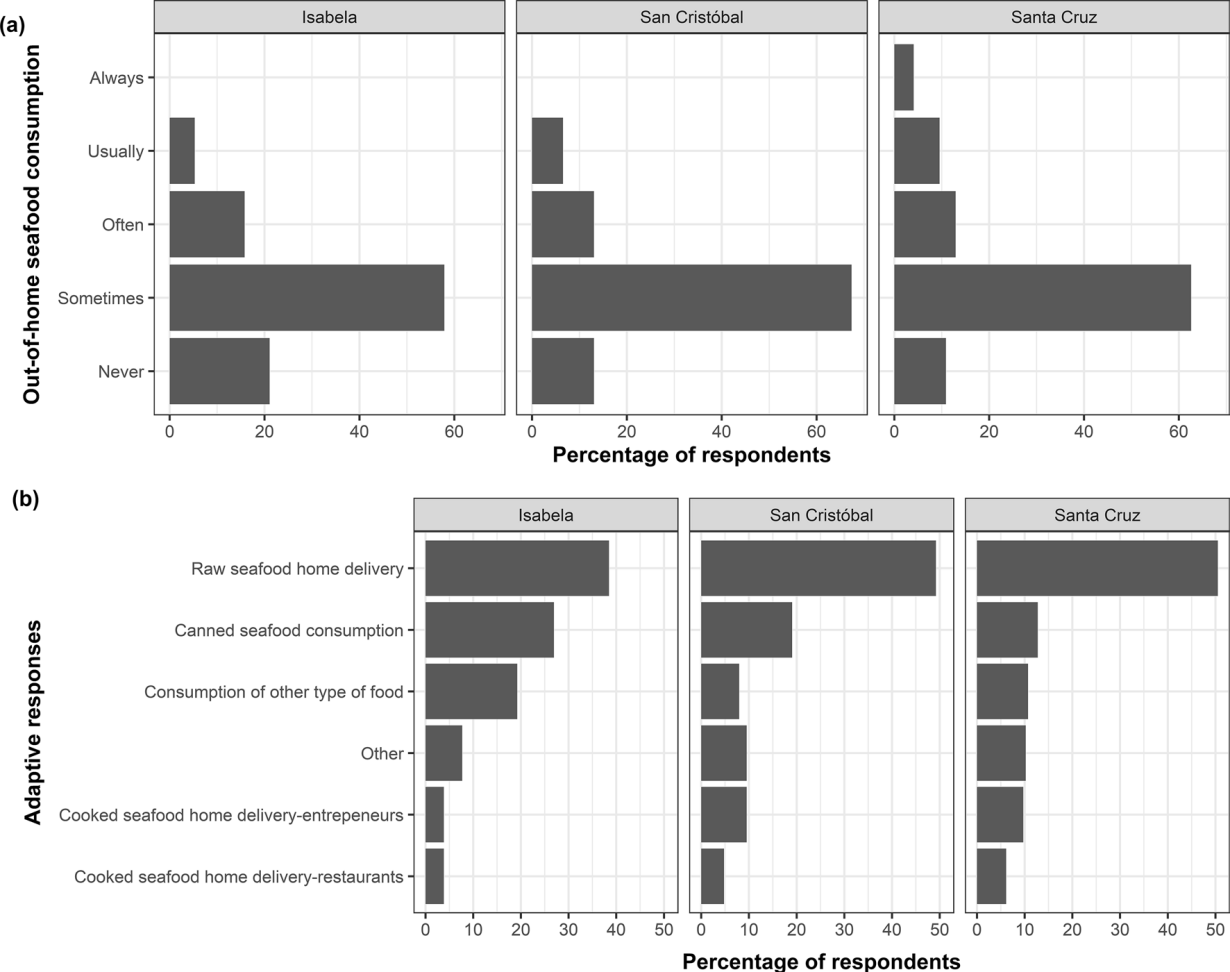
Seafood consumption habits in Isabela, San Cristobal, and Santa Cruz before and during the lockdown implemented in the Galapagos Islands from March 17th to July 1st, 2020. (**a**) Weekly frequency of out-of-home seafood consumption by Galapagos residents (from Monday to Sunday) before the lockdown; (**b**) adaptive responses used by seafood consumers to face the closure of restaurants occurred during the lockdown.

**Figure 3:**
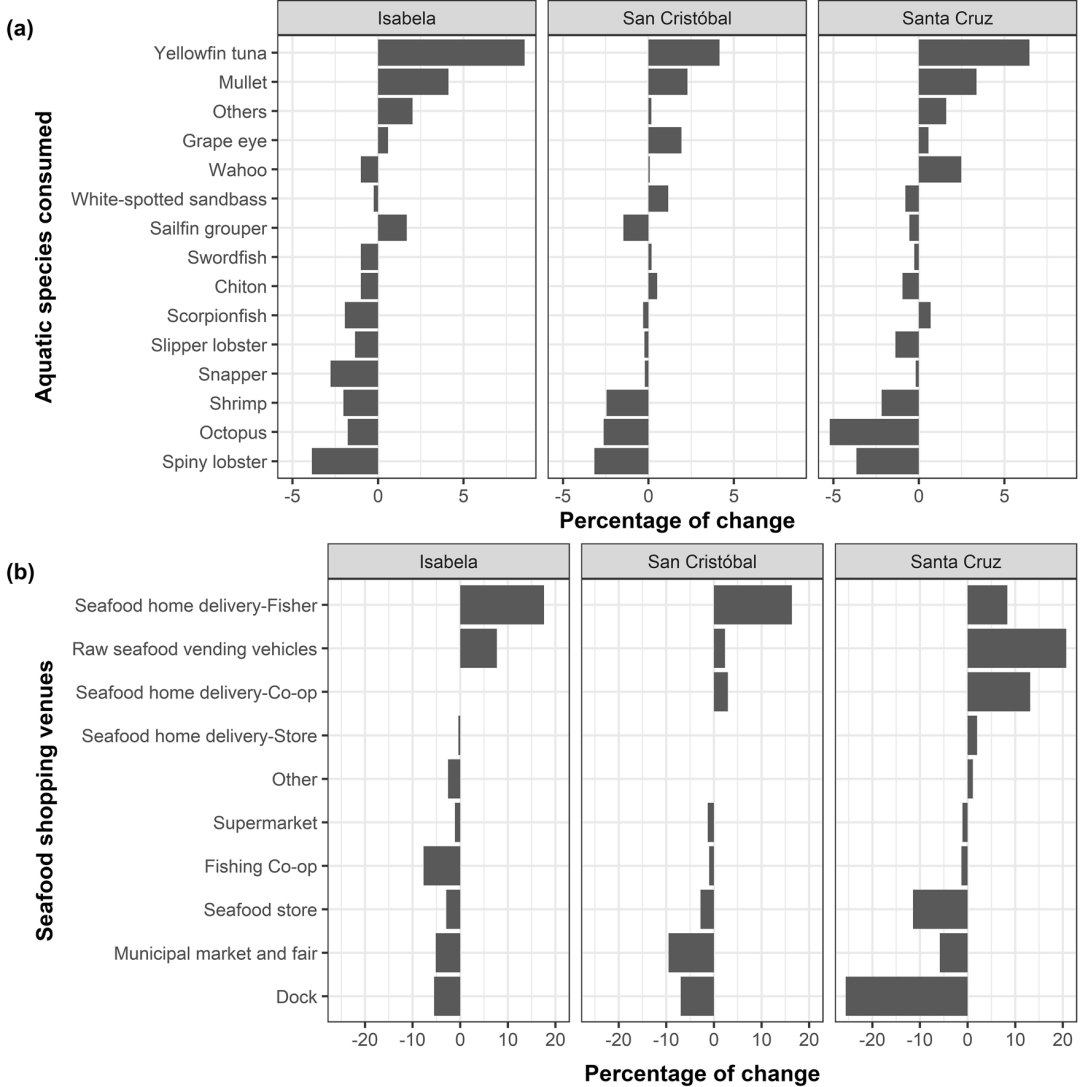
Variations in aquatic species consumed and seafood shopping venues in Isabela, San Cristobal, and Santa Cruz before and during the lockdown implemented in the Galapagos Islands from March 17th to July 1st, 2020. Percentage of change: (**a**) aquatic species consumed; and (**b**) seafood shopping venues.

Before the lockdown, most Galapagos residents commonly dined out, especially on the more populated islands, with over 58% eating seafood out one or two days per week, and more than 21% doing so more than three days per week (Fig. 2a). In response to restaurant closures during the lockdown, residents adapted their seafood consumption habits. Home delivery of fresh/frozen seafood became a prevalent adaptive response, with up to 51% of residents in more populated islands like Santa Cruz choosing this option (Fig. 2b). The consumption of canned seafood also showed an increase, especially on less populated islands like Isabela, where it reached 27% (Fig. 2b). Additionally, on Isabela, 19% of residents opted for different types of food, while 8% chose cooked seafood delivery services. In Santa Cruz and San Cristobal, less than 11% chose other food types, and over 13% opted for cooked seafood delivery from restaurants and entrepreneurs (Fig. 2b). The lockdown prompted other changes in the seafood consumption patterns of Galapagos residents, including modifications in the types of aquatic species consumed, seafood purchasing venues, and factors influencing purchasing decisions. Before the lockdown, the most consumed aquatic species by respondents included yellowfin tuna (18%), imported shrimp (12%), scorpionfish (11%), octopus (10%), sailfin grouper (10%), and wahoo (10%) (Fig. S3). During the lockdown, Galapagos residents increased slightly the consumption of yellowfin tuna, mullet (*Mugil cephalus*), and grape-eye seabass (*Hemilutjanus microphthalmos*) (Fig. 3a, Fig. S3), highlighting the importance of these species for the food security and the economy of Galapagos during times of crises. In contrast, the consumption of crustaceans and mollusks declined across the islands (Fig. 3a, Fig. S3).

Before the lockdown, Galapagos residents used to purchase seafood from docks, seafood stores, municipal markets and fairs, and fishing cooperatives, particularly in Santa Cruz and San Cristobal. However, during the lockdown, there was a transition towards seafood home delivery services across islands (Fig. 3b). For instance, in Santa Cruz, a local fishing cooperative using a refrigerated truck for door-to-door deliveries emerged as a prevalent distribution channel, while seafood home delivery by individual fishers was more popular in San Cristobal and Isabela (Fig. 3b).

**Figure 4:**
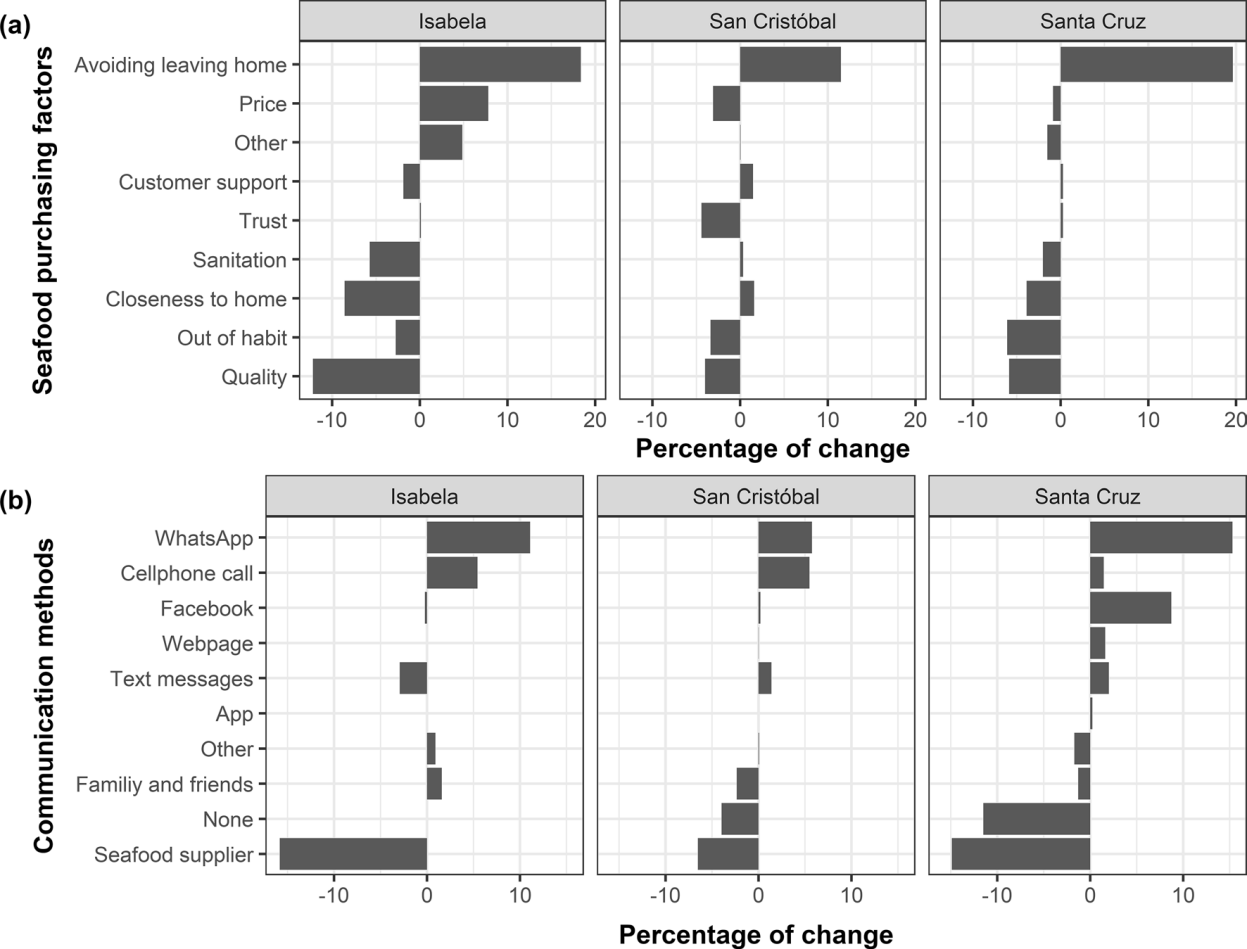
Variations in the factors influencing customer seafood purchases and the communication methods used by seafood consumers in Isabela, San Cristobal, and Santa Cruz, before and during the lockdown implemented in the Galapagos Islands from March 17th to July 1st, 2020. Percentage of change in: (**a**) seafood purchasing factors; (**b**) communication methods.

Before the COVID-19 pandemic, quality was the predominant factor influencing seafood purchases on the three islands (Fig. 4a). However, a reevaluation of priorities occurred during the lockdown, with avoiding leaving home becoming a more significant consideration in purchasing decisions, especially in Isabela and Santa Cruz (Fig. 4a). Even though quality remained crucial, its importance, along with other factors like supplier trust, habitual seafood purchasing venues, and price demonstrated a relative decline in Santa Cruz and San Cristobal. A similar trend was observed in Isabela, except for price, which increased in importance by 8%. (Fig. 4a).

The lockdown also triggered a notable shift in communication strategies related to seafood marketing in the Galapagos. Residents adapted to the mobility restrictions during the lockdown by modifying their seafood information sourcing methods. Traditional reliance on seafood suppliers and personal networks like family and friends declined, giving way to an increase in digital communication (Fig. 4b). Platforms such as WhatsApp and cellphone calls showed a marked uptick in usage, becoming more central in the local information-sharing network in the three islands. Particularly in Santa Cruz, there was a diversification in communication channels, with residents increasingly using platforms such as Facebook, webpages, and text messages (Fig. 4b). Despite the diversification in communication strategies, e-commerce apps remained on the periphery, failing to gain significant popularity across the islands during the lockdown (Fig. 4b).

### Lockdown impact on food security

**Figure 5:**
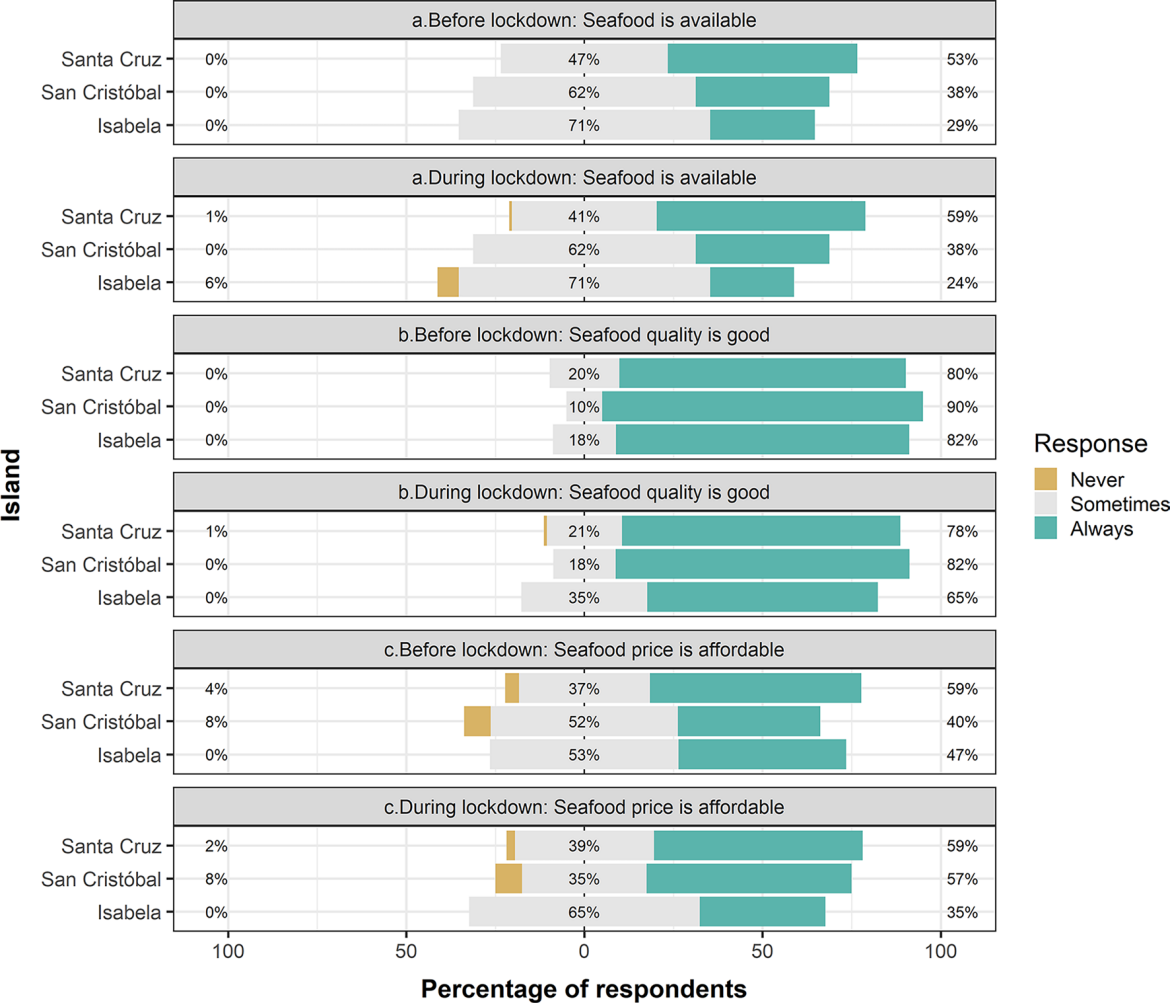
Variations in seafood availability, quality, and affordability in Isabela, San Cristobal, and Santa Cruz, before and during the lockdown implemented in the Galapagos Islands from March 17th to July 1st, 2020.

The lockdown exacerbated a pre-existing seafood insecurity in the Galapagos, denoted by limited physical, social, and economic access to seafood, particularly in less populated islands. Only 53% of Santa Cruz residents reported always having access to seafood before the lockdown, while the same was true for only 38% and 29% of inhabitants of San Cristobal and Isabela, respectively (Fig. 5). Seafood affordability followed a similar trend, with 59% of Santa Cruz participants asserting that seafood prices were always affordable before the lockdown, decreasing to 47% and 40% for participants from Isabela and San Cristobal, respectively. Remarkably, the subjective quality of seafood was favorably rated by over 80% of participants across all islands, before the lockdown (Fig. 5).

The lockdown brought about a mixed bag of impacts on seafood availability, affordability, and quality across islands. For instance, while seafood availability slightly improved in Santa Cruz, and it remained stable in San Cristobal, the opposite trend was noticed in Isabela (Fig. 5). Seafood affordability also showed diverging trends, while it improved notably in San Cristobal, and remain stable in Santa Crus, Isabela witnessed a decline. Meanwhile, the perceived quality of seafood remained stable in Santa Cruz, but declined in Isabela and San Cristobal, during the lockdown (Fig. 5).

### Potential persistence of seafood consumption habits post-lockdown

**Figure 6:**
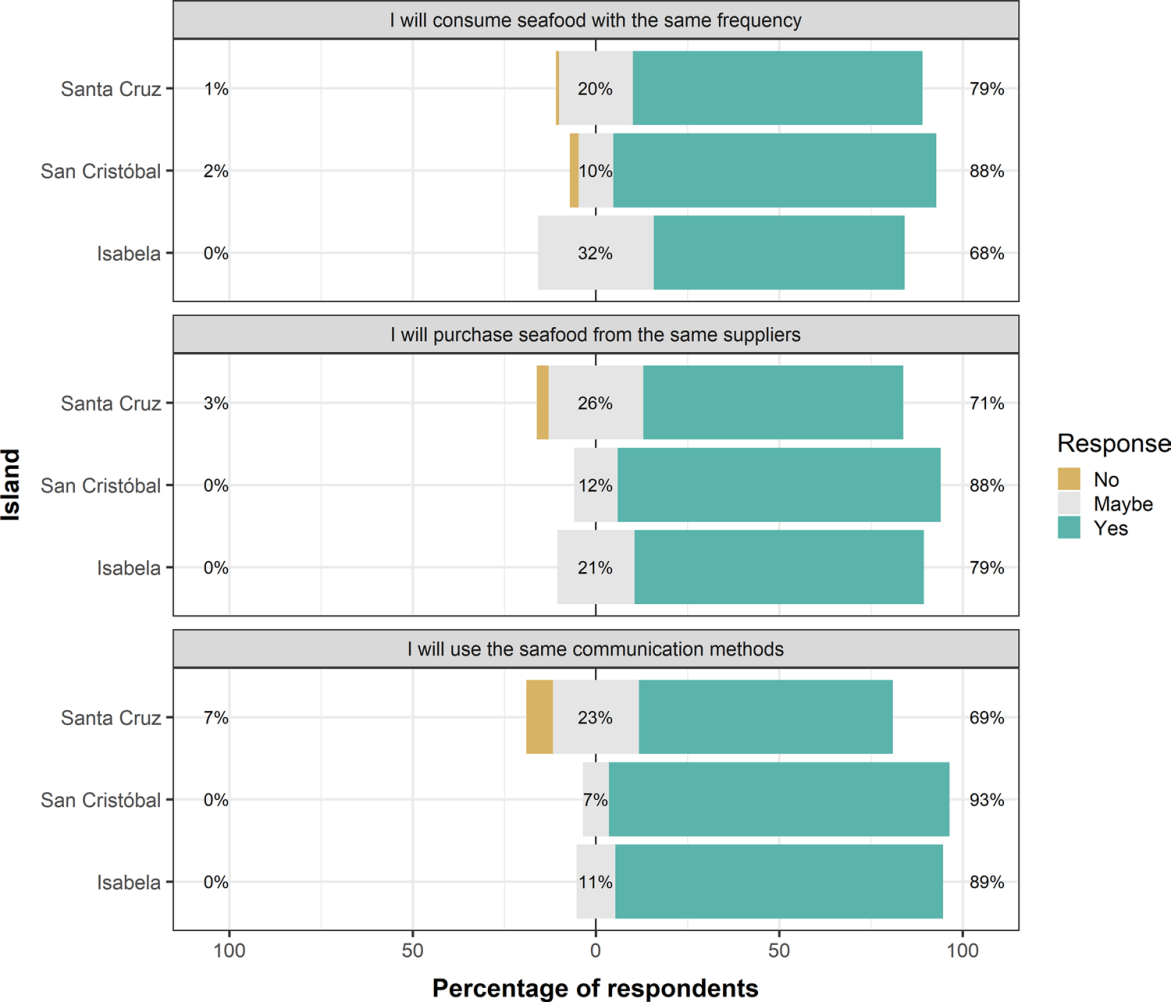
Intention of Galapagos residents to continue the seafood consumption habits adopted during the lockdown in Isabela, San Cristobal, and Santa Cruz after the conclusion of the lockdown implemented in the Galapagos Islands from March 17th to July 1st, 2020.

Our findings indicate a strong intention among Galapagos residents to keep the seafood consumption habits they adopted during the lockdown. A high percentage of participants from Santa Cruz (71%), San Cristobal (88%), and Isabela (79%) expressed their intention to keep purchasing seafood from their existing suppliers. Additionally, a notable proportion of participants (68–93%) across all islands aim to sustain their rate of seafood consumption, and communication methods with suppliers, after the lockdown concludes. San Cristobal showed the highest inclination towards maintaining these habits (Fig. 6).

### Drivers of seafood consumption

**Figure 7:**
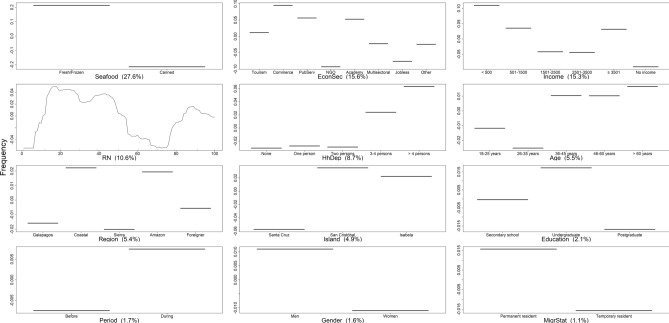
Variation in the percentage of seafood consumption by residents in relation to predictor variables, before and during the lockdown implemented in the Galapagos Islands from March 17th to July 1st, 2020, as predicted by the Boosted Regression Tree (BRT) model. The response variable (Frequency) has been centered by subtracting its mean. Variable importance (VI) scores are shown in parentheses. *RN* random number. Response variables with VI scores exceeding RN represent key predictors of the frequency of seafood consumption by residents in the Galapagos Islands.

Seafood type, economic sector, and individual monthly income were identified by the BRT model as the principal predictors affecting seafood consumption frequency in Galapagos, contributing 58.5% of the VI score (Fig. 7; Table S3). The remaining predictors were not relevant to predict seafood consumption frequency, as they performed worse than RN. These results indicate that the lockdown did not influence seafood consumption frequency, despite increasing rates of consumption during such a period (Fig. 7). The BRT model explained 63% of the deviance in the data, while Pearson’s correlation coefficient was 0.6 (Table S3), highlighting its predictive accuracy. Partial dependence plots indicated that fresh and frozen seafood were preferred by Galapagos residents over canned seafood, while commerce, government, tourism, and academia were the economic sectors with the highest rates of seafood consumption (Fig. 7). In contrast, the sectors that consumed seafood the least frequently were jobless residents and NGO. Notably, individuals earning less than US$ 500 or more than US$ 3501 monthly showed higher consumption rates, while those without an income or earning between US$ 1500 and US$ 3501 consumed seafood less frequently (Fig. 7).

**Figure 8:**
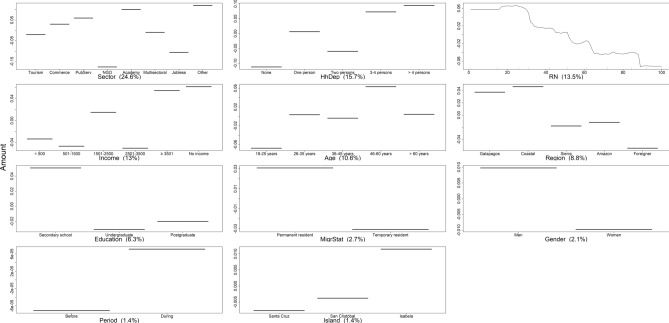
Variation in the percentage of the amount of seafood consumed by residents in relation to predictor variables, before and during the lockdown implemented in the Galapagos Islands from March 17th to July 1st, 2020, as predicted by the boosted regression tree (BRT) model. The response variable (amount) has been centered by subtracting its mean. Variable importance (VI) scores are shown in parentheses. *RN* random number. Response variables with VI scores exceeding RN represent key predictors of the amount of seafood consumed by residents in the Galapagos Islands.

Economic sector and number of household dependents were also identified by the BRT model as key predictors influencing the average weekly seafood consumption in the Galapagos, contributing 40% to the VI score (Fig. 8; Table S4). Other variables had minimal impact on consumption patterns, as they performed worse than RN. Contrary to expectations, the lockdown did not influence the overall quantity of seafood consumed in Galapagos, despite a noticeable increment during this period. (Fig. 8). In this case, the BRT model explained 56% of the deviance in the data, while the Pearson correlation coefficient was 0.5, indicating a robust predictive performance (Table S4). Partial dependence plots indicated diverse weekly seafood consumption patterns among economic sectors. Those in unspecified sectors, academia, public services, and commerce consumed higher amount of seafood, while jobless residents and NGOs consumed the least. Furthermore, households with more dependents generally consumed more seafood, with an exception being observed in households with two dependents, who consumed less compared to those with a single dependent (Fig. 8).

## Discussion

The COVID-19 pandemic exposed the strong vulnerability of the Galapagos tourism-based economy to global socioeconomic perturbations. Nevertheless, the seafood system displayed moderated resilience to the pandemic’s socioeconomic disruptions, thanks to the adaptive capacities of seafood consumers and seafood suppliers. A variety of adaptive responses were adopted by Galapagos residents to cope with the lockdown, which were shaped by socioeconomic factors and the capacity of seafood suppliers to remain competitive in a constricted market. Consumers modified their seafood consumption habits, while fishers adapted their harvesting and marketing strategies. These interrelated adaptive responses represent a valuable opportunity to foster a systemic transformation of the Galapagos seafood system to enhance its resilience against future crises caused by new pandemics, climate change, or other natural and anthropogenic drivers of change.

### Seafood consumption patterns and adaptive responses during the lockdown

The COVID-19 pandemic’s lockdown brought about shifts in Galapagos residents’ seafood consumption habits. Confronted by the closure of the main seafood purchasing venues, seafood consumers and suppliers resorted to the following six adaptive responses to face the lockdown.

1. Shifting procurement venues: the lockdown forced Galapagos residents to change their seafood procurement habits due to the closure of the main seafood purchasing venues. Therefore, they sought alternative places to purchase or obtaining access to seafood, creating the opportunity for the emergence of “alternative seafood networks”, such as home delivery services and a barter economy. These alternative seafood networks represent individual and collective efforts by seafood suppliers, including fishers, fishing families, and retailers to establish direct connections with consumers through new marketing strategies^50^, fostering a network of resilience in the face of adversity.
2. Innovation in seafood marketing strategies: seafood suppliers, including fishers and entrepreneurs, embraced innovative marketing strategies, such as door-to-door seafood home delivery services, to ensure the continued distribution of fishery products within their communities^18^^.^ Local fishing cooperatives like COPROPAG pioneered this model in response to the oversupply of fresh and frozen fish in the local market, attributed to the collapse of the tourism industry and disruptions in commercial flights during the lockdown. Key assets such as a refrigerated truck, coupled with a robust community network, were instrumental for the successful implementation of this innovative model. In contrast, San Cristobal and Isabela, where there was a limited presence of collective assets and a lesser propensity for community-wide actions, individual rather than collective adaptive responses were observed. Independent fishers and entrepreneurs engaged in direct seafood home deliveries, employing a diverse range of transportation methods, such as bicycles, motorcycles, and taxis^18^.
3. Modification of dietary habits: during the lockdown, residents, especially in less populated areas like Isabela, adapted their diets due to fluctuating seafood availability. Limited access to fresh and frozen seafood, driven by reduced supply in Isabela, led to increased consumption of canned seafood and non-seafood items. Factors such as convenience, long shelf life, and lower cost of canned seafood made it an attractive option for residents that faced financial hardships during the lockdown. Furthermore, the early phase of the pandemic probably led to panic buying, driving a preference for non-perishable items. Additionally, the varied cultural backgrounds of residents might have also influenced food choices, leading to a higher reliance on proteins like chicken and meat, reflecting an integration of culinary tradition and emerging necessities in dietary adaptations.
4. Diversification of consumed aquatic species: the dietary preferences of Galapagos residents, traditionally inclined towards the consumption of "whitefish" species such as the sailfin grouper^51^, experienced a shift during the lockdown, leading to a broader diversification in the consumption of aquatic species. During the lockdown, the consumption of yellowfin tuna increased by 6% (Fig. 3a, Fig. S3), consolidating its ranking as the most consumed species by Galapagos residents. This shift in consumption patterns could be attributed to an oversupply of yellowfin tuna during the lockdown, whose landings increased from 89.5 t in 2019 to 165.6 t in 2020^52^. The resulting local market saturation caused a reduction in tuna prices from $US3.50 per pound to $US2.50 per pound during the lockdown^18^. Economic hardships faced during the pandemic probably induced consumers towards more economic seafood choices, leading to increasing slightly the consumption of tuna and other lesser-known, economic species, such as mullet and grape-eye seabass. These findings emphasize the key role of these species in sustaining food security and supporting the local economy in times of crisis. Conversely, the consumption of crustacean and mollusks, particularly of shrimps, decreased across the islands, likely due to their reduced availability and higher prices. The preference for more accessible and affordable aquatic species during the lockdown may have broader implications for the recovery of overexploited demersal finfish fisheries in Galapagos^53^, such as the sailfin grouper and mottled scorpionfish^54^. The landings of these species declined during the lockdown, with scorpionfish landings decreasing from 46.3 t in 2019 to 14.8 t in 2020^52^. These figures suggest a potential reduction in fishing pressure, although further research is required to test this hypothesis.
5. Recalibration of seafood purchasing factors: before the pandemic, seafood purchases in Galapagos primarily depended on species type and quality^51^. However, residents recalibrated these seafood purchasing factors to adapt to the new market dynamics caused by the pandemic. Quality continued to be the main factor influencing seafood purchasing decisions, in alignment with pre-pandemic behaviors, valuing aspects such as taste, freshness, and overall product appeal^51^. Trust in suppliers and geographical proximity also remained significant considerations during the buying process. However, the lockdown introduced new seafood purchasing factors. The need to minimize exposure to COVID-19, pushing consumers towards seafood home delivery options. While price was not a dominant consideration in pre-pandemic purchasing habits, the economic crisis caused by the COVID-19 pandemic escalated its relevance, particularly in areas like Isabela, where limited availability intensified price sensitivities. This change highlighted the economic considerations induced by the pandemic, marking a shift in consumer priorities and adaptation to the evolving seafood market landscape.
6. Adaptation of communication methods: before the lockdown, seafood availability information was obtained through seafood suppliers, family, friends, or WhatsApp. In Santa Cruz, however, residents primarily purchased seafood directly at the docks, seafood stores, or fishing cooperatives, without heavily relying on communication platforms like WhatsApp because the availability of seafood is usually consistent. However, to ensure uninterrupted access to seafood supply, residents, fishers, and cooperatives of Santa Cruz, San Cristobal and Isabela diversified their communication methods during the lockdown. Utilizing online platforms like WhatsApp and Facebook, seafood consumers and suppliers enhanced their connectivity and information flow regarding seafood availability and purchase options, enhancing the marketing and distribution efficiency of seafood throughout the archipelago during the lockdown.

The six adaptive responses described reflect the adaptive capacity of the Galapagos seafood system to the unprecedented challenges provoked by the COVID-19 pandemic. Each adaptive response was shaped by each inhabited island’s unique socioeconomic dynamic and the adaptive capacities of fishers, fishing cooperatives, and entrepreneurs, who were able to shift from a tourism and export-oriented market to a resident and domestic-oriented one. Such transition was also reported in Indonesia, India, Peru, and the United States, where local and direct seafood sales emerged as a critical adaptive strategy in the early stages of the pandemic^1,50^.

### Lockdown impact on seafood security

Our findings suggest that Galapagos residents were experiencing seafood insecurity before the lockdown, especially in Isabela, where residents reported low seafood availability, quality, and affordability. This situation deteriorated during the lockdown, causing residents to increase their consumption of canned seafood and other types of food, likely as an adaptive response to scarcity, unaffordability, and poor quality of fresh and frozen seafood. Conversely, in Santa Cruz, seafood quality and affordability remained relatively stable, while availability slightly increased, presumably due to an oversupply of fresh and frozen fish resulting from restaurant closures and the halt of commercial flights to mainland Ecuador. In this context, the efforts of COPROPAG, independent fishers, and entrepreneurs in delivering seafood aided residents in maintaining access to this essential source of protein during the lockdown.

In San Cristobal, seafood availability remained constant, while affordability increased, and quality decreased during the lockdown. Given that San Cristobal has fewer residents, tourists, and restaurants than Santa Cruz, the local seafood market is smaller. However, the number of fishers in San Cristobal is the highest in the Galapagos, probably resulting in a highly competitive local market. Since both fishing cooperatives and independent fishers faced constraints in exporting fish to mainland Ecuador due to the disruption of commercial flights, competition among seafood suppliers possibly escalated, leading to lower seafood prices to attract more customers. This scenario might also have been influenced by an oversupply in the local market due to disrupted demand and logistical challenges. Our results suggest that this adaptive response made seafood more affordable for residents. Yet, our findings also imply that such competition and oversupply might have led to a decrease in quality. Such decline might stem from various factors, including cost-cutting measures by fishers or challenges in maintaining the cold chain due to inadequate infrastructure and resources among fishing cooperatives and independent fishers in San Cristobal and Isabela. Additionally, the pressure of managing an unexpected surplus of perishable goods could have contributed to quality reduction. To validate these hypotheses and achieve a comprehensive understanding of the market dynamics during the lockdown, further research is necessary. This research should focus on the experiences and perspectives of those directly involved in the seafood industry in the Galapagos Islands including fishers, intermediaries, and fishing cooperatives.

### Drivers of seafood consumption

Contrary to expectations, the COVID-19 pandemic did not have a remarkable influence on the frequency and amount of seafood consumed in the Galapagos. It was anticipated that residents would increase their seafood consumption during the lockdown due to restaurant closures and an oversupply of fresh and frozen seafood, resulting from the collapse of the tourism industry and the disruption of commercial flights. However, the BRT models revealed that the economic sector to which Galapagos residents belong, rather than the lockdown, was the main predictor shaping seafood consumption patterns. Residents working in commerce, tourism, and academic sectors consumed seafood more frequently and in larger quantities during the lockdown, while jobless and those in NGO consumed the smallest amount of seafood with the least frequency. This pattern aligns with findings by Rahman et al.^55^, who found that public sector employees consume more fish than students, self-employed individuals, and professionals from the private sector in Bangladesh. On the other hand, Farmery et al.^56^ found that jobless people are less likely to consume seafood due to the perception of it being expensive. However, these consumption patterns are not universally applicable, as shown by Yadav et al.^57^, who found that profession did not significantly influence fish consumption habits in the Maldives. These studies indicate the variability of occupational influence on seafood consumption among different regions and cultures.

In the Galapagos context, residents' occupation probably influences their seafood consumption patterns due to factors such as accessibility to fresh seafood, environmental concerns, and economic condition. These factors probably generated variations in seafood consumption patterns based on the particular features of the economic sector they belong to. For instance, residents employed in restaurants, hotels, and tourist cruises, such as chefs, waitresses, and naturalist guides, may have greater access to fresh seafood and, therefore, may consume it more frequently. On the other hand, some residents working in NGOs perhaps avoid seafood consumption due to environmental concerns related to overfishing and the negative impact of unsustainable fishing practices on marine ecosystems. Furthermore, economic factors, like the average monthly income of each economic sector, could impact seafood consumption rates. However, cultural backgrounds and personal values could further shape seafood consumption patterns^30^. Therefore, further research is required to understand how the socioeconomic and cultural features that distinguish each Galapagos’ economic sector shape seafood consumption habits in the archipelago.

Seafood type and individual monthly income also played crucial roles in consumption habits during the lockdown. Residents exhibited a preference for fresh and frozen seafood over canned variants. BRT models also revealed paradoxical consumption patterns, such as higher consumption rates among the wealthiest and the least affluent residents. These findings are contrary to Mandal et al.^58^, who found a positive correlation between wealth and seafood consumption rates in Bangladesh. In Galapagos, the wealthiest group could comfortably afford seafood more frequently, while less affluent residents likely engaged in subsistence fishing or barter economies, exchanging goods or services for seafood. Additionally, a significant drop of 29% in fish prices during the lockdown may have enhanced the affordability of seafood for the least affluent residents^18^, who probably also received food kits delivered by governmental institutions or fish donated by fishers, enabling them to increase their seafood consumption rates.

Middle-income residents, the jobless, and those maintaining formal employment statuses without actual income, like naturalist guides, were found to consume seafood less frequently. One plausible explanation among middle-income individuals could be a preference for consuming seafood in restaurants rather than at home, a behavior reported by previous studies^32^. During the lockdown, with restaurants closed, this demographic group probably opted for alternative, more convenient food choices that required less preparation time. In contrast, the jobless faced financial constraints, limiting their ability to purchase seafood, thus reducing their consumption rates. Naturalist guides, while formally employed, encountered financial hardships due to the pandemic-induced cancellations of cruises, compelling them to choose more affordable, potentially non-seafood dietary options. This group might also have experienced psychological impacts due to the discrepancy between apparent employment stability and actual financial instability, further influencing their consumption patterns and food choices during the lockdown. Thus, the divergence trends between low-income individuals, who consumed more seafood, and jobless individuals, who consumed less, might be attributed to low-income individuals' engagement in subsistence fishing or barter economies, allowing higher seafood consumption despite financial constraints. In contrast, jobless individuals, facing financial limitations and possibly perceiving seafood as a luxury, tended to consume it less. This difference highlights the complex dynamics of socio-economic status in food consumption patterns during the lockdown.

Lastly, our study revealed that the number of household dependents also played a significant role in seafood consumption patterns, with larger household dependents, probably those with children or elderly dependents, consuming more seafood. This correlation can vary, based on the household members' age composition^59^. For example, Abusin et al.^60^ found that households with more people tend to consume less fish, while households with children tend to consume more fish^60^.

In summary, the adaptive responses triggered by the lockdown have created opportunities to boost the health and nutrition of Galapagos residents, mitigate the environmental impact of fishing on over-exploited demersal finfish fisheries, and enhance the livelihoods of small-scale fishers through changes in seafood consumption habits. Nevertheless, further research is needed to explore a broader spectrum of socio-cultural, situational, psychological, biological, and physiological factors—as suggested by Saidi et al.^59^—to comprehensively understand the complexity of seafood consumption patterns in the Galapagos.

### Opportunities for a systemic transformation of the Galapagos seafood system

Our survey reveals a moderate adaptive capacity among Galapagos seafood consumers, fishers, and fishing cooperatives, in response to the disruptions caused by the COVID-19 pandemic. The observed adaptive responses appear to be more a pragmatic and reactive response to an immediate crisis rather than being part of a pre-existing strategic or contingency plan to transform customary practices. Thus, adaptations in consumer behavior and seafood marketing strategies may represent a circumstantial necessity rather than a genuine willingness to improve the resilience of the Galapagos seafood system. Therefore, continual promotion of sustainable consumption and responsible fishing practices is essential as residents navigate the challenges and opportunities presented beyond the pandemic. This insight becomes increasingly relevant as Galapagos experiences a significant resurgence in tourism, escalating to concerning historical peaks^61^.

The inclination of Galapagos residents to maintain the seafood consumption habits adopted during the pandemic represents a valuable opportunity to promote a systemic transformation of the Galapagos seafood system. Pursuing this objective, we recommend promoting fish consumption to enhance the health and nutrition of Galapagos residents through educational campaigns that elucidate the benefits of fish consumption. This initiative should be complemented with strategies to simplify seafood preparation at home, such as cooking classes and the introduction of easy-to-prepare seafood products. Nevertheless, eco-friendly packaging should be emphasized during the development of new products to prevent potential adverse ecological impacts.

The consumption of pelagic species, particularly of yellowfin tuna, wahoo, and grape-eye seabass, should be prioritized to alleviate the pressure on overexploited demersal finfish and, thus, support the recovery of the ecological role these species play in the Galapagos marine ecosystems. Enhancing consumption of the pelagic species will also bolster the archipelago’s food security by lessening dependence on imported foods. This objective can be facilitated through targeted communication and marketing campaigns, designed to reshape seafood consumption habits by considering the key factors that influence the purchasing decisions of Galapagos residents. Such campaigns should include information on the recommended maximum weekly intake of species like yellowfin tuna given its high concentration of mercury, zinc, and cadmium^62,63^. Furthermore, the organization of gastronomic events in partnership with fishers, restaurants, and retailers could showcase the taste, quality, and culinary versatility of tuna and other pelagic finfish species. Additionally, the development of value-added products, such as ready-to-eat meals, snacks, and supplements, can increase their appeal and accessibility, further encouraging their consumption. Improving processing and packaging methods will enhance the quality, shelf-life, and convenience of these seafood products, promoting diversification in the local economy and increasing adaptive capacities through alternative revenue streams. Moreover, the sustainable development of a local small-scale tuna offshore fishery could supply the local market of higher quality tuna. The same strategy has been suggested by Dacks et al.^64^ as an alternative to mitigate the socioeconomic impact generated by the establishment of the Palau National Marine Sanctuary (PNMS), a large marine protected area where fishing is only allowed in ca. 20% of Palau’s Exclusive Economic Zone (EEZ). In this Small Island Developing State, as in the GMR, significant investments in science, technology, and innovation are needed to promote the transition from coastal fisheries towards a small-scale tuna offshore fishery^53,64^.

As yellowfin tuna is a key species to enhance the food security and economy of Galapagos, we suggest adopting a strategic, comprehensive, and collaborative approach to address the numerous barriers that precludes the supply of high-quality tuna in a sustainable way. Challenges such as illegal, unreported, and unregulated (IUU), fishing market fragmentation, cold-chain inadequacies, lack of quality differentiation, and external price dependencies hinder the enhancement of the tuna market and its appeal to premium sectors, including high-end tourism^40^. To address these challenges, it is advised to extend technical and financial support to fishers and entrepreneurs, facilitating the emergence and growth of sustainable and socially responsible seafood enterprises. Following Viteri et al.^65^ and Castrejón et al.^66^, we propose the establishment of a seafood innovation lab to assist fishers and entrepreneurs in refining processes related to the capture, handling, freezing, processing, and marketing of tuna and other pelagic fish. The lab would provide specialized training and technical advice to enhance local capacities to recognize and handle high-grade tuna, as well as for the development of fishing ventures, the production of value-added products, and the marketing of seafood products in local, national, and international markets. The goal is to foster a market that supports responsible seafood consumption and assist seafood suppliers in locating and gaining access to markets that provide fair prices for high-quality, socially responsible, and environmentally sustainable seafood products. Nevertheless, to promote responsible fishing practices, the introduction of market incentives such as ecolabeling and certification schemes, alongside incentives encouraging the adoption of advanced monitoring and traceability technologies, is essential^67^. These measures, complemented by performance-based penalties and rewards, could facilitate a gradual improvement in fishing practices, minimizing bycatch and IUU fishing^67^.

To ensure the effective implementation of these recommendations, it is crucial that all initiatives actively involve the most relevant stakeholders within the Galapagos seafood system. This bottom-up approach should include fishers, chefs, restaurant owners, tourist entrepreneurs, and intermediaries, ensuring that the implemented strategies are both culturally relevant and contextually suite. Additionally, collaboration with policymakers, researchers, NGO, and community leaders will be crucial to address systemic barriers and create an enabling environment that catalyzes a transition toward more sustainable seafood consumption and production practices in Galapagos. This systemic transformation is crucial to bolster the resilience of the Galapagos seafood system against future crises stemming from natural and anthropogenic drivers of change.

### Supplementary Information

Supplementary Information.

## Data Availability

The datasets generated during and/or analyzed during the current study are available from the corresponding author on reasonable request.
