# Supplementary material for: The impact of the COVID-19 pandemic on the Galapagos Islands' seafood system from consumers’ perspectives
Source: Sci Rep. 2024 Jan 19;14:1690. doi: 10.1038/s41598-024-52247-5 (PMC10798946; doi:10.1038/s41598-024-52247-5)
Supplement: Supplementary file 1 — Supplementary Information. [file 41598_2024_52247_MOESM1_ESM.docx]

Supplementary Information

The impact of the COVID-19 pandemic on the Galapagos Islands' seafood system from consumers’ perspectives

Mauricio Castrejón*, Jeremy Pittman, Cristina Miño, Jorge Ramírez-González, César Viteri, Nicolas Moity, Solange Andrade-Vera, Renato Caceres, Michael K. Tanner, Gabriela Rodríguez, María José Barragán-Paladines

*** Correspondence:** Mauricio Castrejón: [hugo.castrejon@udla.edu.ec](mailto:hugo.castrejon@udla.edu.ec)

# Supplementary data

## Survey (Spanish version)

Estimada comunidad de Galápagos,

Conscientes de la crisis que enfrenta la Provincia de Galápagos por causa del virus COVID-19, investigadores de la Fundación Charles Darwin y la Universidad de Waterloo (Canadá), bajo el aval del Parque Nacional Galápagos (PNG) y el Consejo de Gobierno del Régimen Especial de Galápagos (CGREG), hemos elaborado una encuesta para obtener información respecto al efecto de la cuarentena del COVID-19 sobre los niveles y patrones de consumo de pescados y mariscos en la Provincia de Galápagos. Por esta razón, solicitamos cordialmente su colaboración para responder esta encuesta de 28 preguntas, cuya duración es de 10 min. La encuesta debe ser contestada únicamente por personas mayores de 18 años, quienes hayan residido en Galápagos, antes y durante la cuarentena establecida desde el 16 de marzo de 2020. Su participación es completamente voluntaria. Si decide participar, tiene la libertad de abandonar la encuesta en cualquier momento o no contestar una o varias de las preguntas. Su participación es anónima.

Los datos colectados serán utilizados para recomendar al Gobierno del Ecuador una estrategia para promover la seguridad alimentaria de Galápagos, así como para el desarrollo de publicaciones científicas. Los resultados de esta investigación serán hechos públicos a través de las redes sociales. Adicionalmente, una vez finalizada la cuarentena, se brindará una presentación virtual y/o pública de los resultados. La fecha y hora de la presentación será dada a conocer próximamente por redes sociales. Esta investigación ha sido revisada y aprobada por un Comité de Ética de la Universidad de Waterloo, bajo el permiso ORE #41662. De antemano agradecemos su colaboración. Cualquier duda o pregunta favor de comunicarse a [hugo.castrejon@udla.edu.ec](mailto:hugo.castrejon@udla.edu.ec) or [jorge.ramirez@fcdarwin.org.ec](mailto:jorge.ramirez@fcdarwin.org.ec).

1. ¿Desea contestar de forma voluntaria esta encuesta?

- Si
- No

Skip To: End of Survey If ¿Desea contestar de forma voluntaria esta encuesta? = No

2. Categoría migratoria

- Residente permanente
- Residente temporal
- Transeúnte
- Turista nacional
- Turista extranjero
- Prefiero no responder

3. Isla de residencia

- San Cristóbal
- Santa Cruz
- Isabela
- Floreana

4. ¿Durante la cuarentena dónde residió?

- Totalmente en Galápagos
- Parcialmente en Galápagos y en el continente
- Totalmente en el continente

Skip To: End of Survey If ¿Durante la cuarentena dónde residió? = Totalmente en el continente

5. Nivel de escolaridad

- Educación básica
- Educación secundaria
- Tercer nivel- Pregrado
- Cuarto nivel- Postgrado
- Ninguna

6. ¿En qué sector trabaja? (seleccione todas las opciones que sean ciertas).

- Sector público
- Turismo
- Comercio
- Transporte
- Agricultura
- Ganadería
- Pesca
- Academia
- ONG
- Desempleado
- Otro ________________________________________________

7. ¿Cuántas personas de su familia dependen económicamente de usted?

- Ninguna
- Una persona
- Dos personas
- 3-4 personas
- Más de 4 personas

8. Por favor ordene del 1 al 4 los alimentos que más consume, siendo "1" el alimento que más consume y "4" el alimento que menos consume.

- ______ Frutas, granos y vegetales
- ______ Carne de res y cerdo
- ______ Pollo
- ______ Pescado y mariscos

9. ¿Con qué frecuencia consume pescado y/o mariscos FRESCOS o CONGELADOS? (seleccione solo UNA opción por cada columna).

|  | ANTES de la cuarentena | DURANTE la cuarentena |
| --- | --- | --- |
|  | Respuesta | Respuesta |
| Nunca consumo pescado y/o mariscos frescos o congelados |  |  |
| Un día al mes |  |  |
| Una vez cada 15 día |  |  |
| Un día por semana |  |  |
| Dos días por semana |  |  |
| Tres días por semana |  |  |
| Más de cuatro días por semana |  |  |
| Diario |  |  |
| Otro |  |  |

10. ¿Con qué frecuencia consume pescado y/o mariscos ENLATADOS? (seleccione solo UNA opción por cada columna).

|  | ANTES de la cuarentena | DURANTE la cuarentena |
| --- | --- | --- |
|  | Respuesta | Respuesta |
| Nunca consumo productos enlatados |  |  |
| Un día al mes |  |  |
| Una vez cada 15 días |  |  |
| Un día por semana |  |  |
| Dos días por semana |  |  |
| Tres días por semana |  |  |
| Más de cuatro días por semana |  |  |
| Diario |  |  |
| Otro |  |  |

11. ¿Qué tipo de pescados y/o mariscos consume con mayor frecuencia? (seleccione todas las opciones que sean ciertas en cada columna).

|  | ANTES de la cuarentena | DURANTE de la cuarentena |
| --- | --- | --- |
|  | Respuesta | Respuesta |
| Atún- albacora |  |  |
| Bacalao |  |  |
| Blanquillo |  |  |
| Brujo |  |  |
| Canchalagua |  |  |
| Camarón |  |  |
| Guajo |  |  |
| Langostino |  |  |
| Langosta |  |  |
| Lisa |  |  |
| Ojo de uva |  |  |
| Pargo |  |  |
| Pez espada |  |  |
| Pulpo |  |  |
| Otros |  |  |

12. ¿Qué cantidad de pescado y/o mariscos CONSUME usted en promedio durante una semana (lunes a domingo)? Especifique la cantidad en libras en cada columna.

|  | ANTES de la cuarentena | DURANTE la cuarentena |
| --- | --- | --- |
|  | Respuesta | Respuesta |
| Media libra o menos |  |  |
| Una libra |  |  |
| Una libra y media |  |  |
| Dos a tres libras |  |  |
| Más de tres libras |  |  |
| No sé |  |  |
| Otro |  |  |

13. Antes de la cuarentena, ¿con qué frecuencia salía a comer fuera de casa durante una semana típica (lunes a domingo)? (restaurantes, kioskos, puestos de comida rápida)

- Nunca (todas mis comidas las realizo en mi casa)
- A veces (uno o dos días por semana)
- Regularmente (3 a 4 días por semana)
- Casi siempre (5 a 6 días por semana)
- Siempre (todos los días salgo a comer fuera de casa)
- Otro

Skip To: 15 If Antes de la cuarentena, ¿con qué frecuencia salía a comer fuera de casa durante una semana típica... = Nunca (todas mis comidas las realizo en mi casa)

14. Considerando que durante la cuarentena los restaurantes han permanecido cerrados, ¿qué ha hecho usted para consumir pescado y/o mariscos? (seleccione todas las opciones que sean ciertas).

- Nada, he preferido consumir otra clase de alimentos (carne, pollo, fruta, o vegetales)
- He comprado pescado y/o mariscos enlatados
- He solicitado comida preparada a domicilio a restaurantes
- He solicitado comida preparada a personas particulares, incluidos pescadores (ceviches, encebollado, etc.)
- He solicitado o comprado pescados y/o mariscos frescos o congelados a domicilio
- Otro ________________________________________________

15. ¿Qué cantidad de pescado y/o mariscos, fresco o congelado, COMPRA usted por SEMANA? (seleccione solo UNA opción por cada columna).

|  | ANTES de la cuarentena | DURANTE la cuarentena |
| --- | --- | --- |
|  | Respuesta | Respuesta |
| Nunca compro pescado y/o mariscos frescos o congelados (los consumo únicamente en restaurantes) |  |  |
| Media libra |  |  |
| Una libra |  |  |
| Una libra y media |  |  |
| Dos libras |  |  |
| Tres libras |  |  |
| Cuatro libras |  |  |
| Más de cuatro libras |  |  |
| Otro |  |  |

Skip To: 25 If ¿Qué cantidad de pescado y/o mariscos, fresco o congelado, COMPRA usted por SEMANA? (seleccione s... : DURANTE la cuarentena = Nunca compro pescado y/o mariscos frescos o congelados (los consumo únicamente en restaurantes) [ Respuesta ]

16. ¿Para quién compra pescado y/o mariscos? (seleccione todas las opciones que sean ciertas en cada columna).

|  | ANTES de la cuarentena | DURANTE la cuarentena |
| --- | --- | --- |
|  | Respuesta | Respuesta |
| Para mi consumo personal |  |  |
| Para el consumo de mi familia |  |  |
| Para mi negocio (restaurante, marisquería, hotel, embarcación, etc.) |  |  |
| Otro |  |  |

17. ¿Dónde compra regularmente su pescado y/o mariscos? (seleccione todas las opciones que sean ciertas en cada columna).

|  | ANTES de la cuarentena | DURANTE la cuarentena |
| --- | --- | --- |
|  | Respuesta | Respuesta |
| En el muelle |  |  |
| En supermercados |  |  |
| En mercado municipal o feria |  |  |
| En cooperativa de pesca |  |  |
| En marisquería |  |  |
| Solicito servicio a domicilio a la cooperativa de pesca |  |  |
| Solicito servicio a domicilio a pescadores particulares |  |  |
| Solicito servicio a domicilio a marisquerías |  |  |
| Compro pescado de los vehículos que ofrecen pescado casa por casa |  |  |
| Otro |  |  |

18. ¿Por qué compra pescado y/o marisco en el lugar de su preferencia? (seleccione todas las opciones que sean ciertas en cada columna).

|  | ANTES de la cuarentena | DURANTE la cuarentena |
| --- | --- | --- |
|  | Respuesta | Respuesta |
| Porque está cerca de mi casa |  |  |
| El pescado y marisco que venden es de buena calidad |  |  |
| Por costumbre |  |  |
| Para evitar salir de casa |  |  |
| Es más barato que en otros lugares |  |  |
| Porque su higiene es buena |  |  |
| Porque me atienden bien |  |  |
| Por confianza |  |  |
| Otro |  |  |

19. ¿Qué medidas ha tomado la persona que regularmente le vende pescado y/o mariscos para evitar la probabilidad de contagio del coronavirus? (seleccione todas las opciones que sean ciertas).

- Tenía una mascarilla puesta correctamente (nariz y boca cubiertas)
- Tenía guantes puestos
- Usó desinfectante para manos antes de manipular el pescado (incluso con guantes puestos)
- La persona que entrega el pescado y/o mariscos es diferente a la persona que cobra el dinero
- Ninguna
- Otro ________________________________________________

20. ¿Cómo se entera regularmente si un vendedor tiene el pescado y/o mariscos que usted desea comprar? (seleccione todas las opciones que sean ciertas en cada columna).

|  | ANTES de la cuarentena | DURANTE la cuarentena |
| --- | --- | --- |
|  | Respuesta | Respuesta |
| Whatsapp |  |  |
| Aplicación web |  |  |
| Mensaje de texto |  |  |
| Llamada por celular |  |  |
| Página web |  |  |
| Facebook |  |  |
| Le pregunto a mi proveedor directamente en persona |  |  |
| Por aviso de familia o amigos |  |  |
| Ninguno |  |  |
| Otro |  |  |

21. ¿Cómo ha afectado la cuarentena los precios de pescados y/o mariscos?

- Disminuyeron mucho
- Disminuyeron poco
- Los precios son los mismos, no han cambiado
- Aumentaron poco
- Aumentaron mucho
- Otro ________________________________________________

22. Por favor seleccione la opción que mejor refleja su opinión, considerando el período ANTES de la cuarentena:

|  | Siempre | A veces | Nunca |
| --- | --- | --- | --- |
| Hay pescado y/o mariscos disponibles cuando lo necesito |  |  |  |
| El pescado y/o mariscos que compro o consumo es de buena calidad |  |  |  |
| El precio del pescado y/o mariscos es accesible para mi economía |  |  |  |

23. Por favor seleccione la opción que mejor refleja su opinión, considerando el período DURANTE de la cuarentena:

|  | Siempre | A veces | Nunca |
| --- | --- | --- | --- |
| Hay pescado y/o mariscos disponible cuando lo necesito |  |  |  |
| El pescado y/o mariscos que compro es de buena calidad |  |  |  |
| El precio del pescado y/o marisco es accesible para mi economía |  |  |  |

24. Por favor seleccione la opción que mejor refleja su opinión, considerando el período POSTERIOR a la cuarentena (es decir, una vez que haya concluido):

|  | Si | Tal vez | No |
| --- | --- | --- | --- |
| Seguiré consumiendo pescado y/o mariscos con la misma frecuencia |  |  |  |
| Seguiré comprando pescado y/o mariscos a los mismos vendedores |  |  |  |
| Seguiré usando los mismos canales de comunicación para saber si hay pescado y/o mariscos disponibles |  |  |  |

25. Género

- Hombre
- Mujer

26. Grupo de edad

- Menor de 18 años
- 18-25 años
- 26-35 años
- 36-45 años
- 46-60 años
- Mayor a 60 años

27. ¿Cuál es su ingreso económico mensual en USD?

- No recibo ningún ingreso por el momento
- Menos de 500
- 501-1000
- 1001-1500
- 1501-2000
- 2001-2500
- 2501-3000
- 3001-3500
- 3501-4000
- 4001-5000
- Más de 5000
- Prefiero no responder

28. Región de nacimiento

- Sierra
- Costa
- Amazonía
- Insular (Galápagos)
- Extranjero
- Otro

## Survey (English version)

Dear Galapagos community,

Being aware of the crisis faced by the Province of Galápagos due to the COVID-19 virus, researchers from the Charles Darwin Foundation and the University of Waterloo (Canada), with the endorsement of the Galápagos National Park (PNG) and the Government Council of the Special Regime of Galápagos (CGREG), have prepared a survey to obtain information regarding the effect of the COVID-19 lockdown on the levels and patterns of consumption of fish and seafood in the Province of Galápagos. For this reason, we kindly ask for your collaboration in answering this 28-question survey, which takes 10 minutes. The survey should be answered only by people over 18 years old, who have lived in Galápagos, before and during the lockdown established since March 16, 2020. Your participation is completely voluntary. If you decide to participate, you are free to abandon the survey at any time or not answer one or several of the questions. Your participation is anonymous.

The data collected will be used to recommend to the Government of Ecuador a strategy to promote food security in the Galapagos, as well as for the development of scientific publications. The results of this research will be made public through social media. Additionally, once the lockdown is over, a virtual and/or public presentation of the results will be provided. The date and time of the presentation will be announced soon through social media. This research has been reviewed and approved by an Ethics Committee of the University of Waterloo, under permit ORE #41662. We appreciate your collaboration in advance. Any questions or doubts, please contact [hugo.castrejon@udla.edu.ec](mailto:hugo.castrejon@udla.edu.ec) or [jorge.ramirez@fcdarwin.org.ec](mailto:jorge.ramirez@fcdarwin.org.ec)

1. Would you like to voluntarily answer this survey?

- Yes
- No

Skip To: End of Survey If Do you want to voluntarily answer this survey? = No

2. Migratory status

- Permanent resident
- Temporary resident
- Transient
- National tourist
- Foreign tourist
- Prefer not to answer

3. Island of residence

- San Cristobal
- Santa Cruz
- Isabela
- Floreana

4. Where did you reside during the lockdown?

- Entirely in Galápagos
- Partially in Galápagos and on the mainland
- Entirely on the mainland

Skip To: End of Survey If Where did you reside during the lockdown? = Entirely on the mainland

5. Education level

- Basic education
- Secondary education
- Undergraduate
- Postgraduate
- None

6. In what sector do you work? (select all that apply).

- Public sector
- Tourism
- Commerce
- Transportation
- Agriculture
- Livestock
- Fishing
- Academia
- NGO
- Unemployed
- Other ­­_________________________________

7. How many people in your household depend on you financially?

- None
- One person
- Two people
- 3-4 people
- More than 4 people

8. Please rank from 1 to 4 the foods you consume the most, with "1" being the food you consume the most and "4" the food you consume the least.

______ Fruits, grains, and vegetables

______ Beef and pork

______ Chicken

______ Fish and seafood

9. How often do you consume FRESH or FROZEN fish and/or seafood? (select only ONE option per column).

|  | BEFORE the lockdown | DURING the lockdown |
| --- | --- | --- |
|  | Answer | Answer |
| Never consume fresh or frozen fish and/or seafood |  |  |
| One day a month |  |  |
| Once every 15 days |  |  |
| One day a week |  |  |
| Two days a week |  |  |
| Three days a week |  |  |
| More than four days a week |  |  |
| Daily |  |  |
| Other |  |  |

10. How often do you consume CANNED fish and/or seafood? (select only ONE option for each column).

|  | BEFORE the lockdown | DURING the lockdown |
| --- | --- | --- |
|  | Answer | Answer |
| Never consume canned products |  |  |
| One day a month |  |  |
| Once every 15 days |  |  |
| One day a week |  |  |
| Two days a week |  |  |
| Three days a week |  |  |
| More than four days a week |  |  |
| Daily |  |  |
| Other |  |  |

11. What type of fish and/or seafood do you consume most often? (select all options that are true in each column).

|  | BEFORE the lockdown | DURING the lockdown |
| --- | --- | --- |
|  | Answer | Answer |
| Yellowfin tuna |  |  |
| Sailfin grouper |  |  |
| White-spotted sandbass |  |  |
| Scorpionfish |  |  |
| Chiton |  |  |
| Shrimp |  |  |
| Wahoo |  |  |
| Slipper lobster |  |  |
| Spiny lobster |  |  |
| Muller |  |  |
| Grape eye |  |  |
| Snapper |  |  |
| Swordfish |  |  |
| Octopus |  |  |
| Others |  |  |

12. What amount of fish and/or seafood do you CONSUME on average during a week (Monday to Sunday)? Specify the amount in pounds in each column.

|  | BEFORE the lockdown | DURING the lockdown |
| --- | --- | --- |
|  | Answer | Answer |
| Half a pound or less |  |  |
| One pound |  |  |
| One pound and a half |  |  |
| Two to three pounds |  |  |
| More than three pounds |  |  |
| I do not know |  |  |
| Other |  |  |

13. Before the lockdown, how often did you eat out during a typical week (Monday to Sunday)? (restaurants, kiosks, fast food stalls)

- Never (I make all my meals at home)
- Sometimes (one or two days a week)
- Often (3 to 4 days a week)
- Usually (5 to 6 days a week)
- Always (I eat out every day)
- Other

Skip To: 15 If Before the lockdown, how often did you eat out during a typical week... = Never (I make all my meals at home)

14. Considering that restaurants have been closed during the lockdown, what have you done to consume fish and/or seafood? (select all true options).

- Nothing, I have preferred to consume other kinds of food (meat, chicken, fruit, or vegetables)
- I have bought canned fish and/or seafood
- I have ordered prepared food for home delivery from restaurants
- I have ordered prepared food from private individuals, including fishermen (ceviches, encebollado, etc.)
- I have ordered or bought fresh or frozen fish and/or seafood for home delivery
- Other ________________________________________________

15. How much fish and/or seafood, fresh or frozen, do you BUY per WEEK? (select only ONE option for each column).

|  | BEFORE the lockdown | DURING the lockdown |
| --- | --- | --- |
|  | Answer | Answer |
| I never buy fresh or frozen fish and/or seafood (I only consume them in restaurants) |  |  |
| Half a pound |  |  |
| One pound |  |  |
| One pound and a half |  |  |
| Two pounds |  |  |
| Three pounds |  |  |
| Four pounds |  |  |
| More than four pounds |  |  |
| Other |  |  |

Skip To: 25 If What amount of fresh or frozen fish and/or seafood do you BUY per WEEK? (select ... : DURING the lockdown = I never buy fresh or frozen fish and/or seafood (I only consume them in restaurants) [ Response ]

16. Who do you buy fish and/or seafood for? (select all that apply in each column).

|  | BEFORE the lockdown | DURING the lockdown |
| --- | --- | --- |
|  | Answer | Answer |
| For my consumption |  |  |
| For my family’s consumption |  |  |
| For my business (restaurant, seafood store, hotel, vessel, etc.) |  |  |
| Other |  |  |

17. Where do you regularly buy your fish and/or seafood? (select all that apply in each column).

|  | BEFORE the lockdown | DURING the lockdown |
| --- | --- | --- |
|  | Answer | Answer |
| At the dock |  |  |
| At supermarkets |  |  |
| At a municipal market and fair |  |  |
| At a fishing cooperative |  |  |
| At a seafood store |  |  |
| I request home delivery from the fishing cooperative |  |  |
| I request home delivery from private fishers |  |  |
| I request home delivery from seafood stores |  |  |
| I buy fish from the seafood vending vehicles |  |  |
| Other |  |  |

18. Why do you buy fish and/or seafood at your preferred place? (select all that apply in each column).

|  | BEFORE the lockdown | DURING the lockdown |
| --- | --- | --- |
|  | Answer | Answer |
| Because it's near my house |  |  |
| The fish and seafood they sell is of good quality |  |  |
| Out of habit |  |  |
| To avoid leaving the house |  |  |
| It is cheaper than other places |  |  |
| Because their hygiene is good |  |  |
| Because they serve me well |  |  |
| Out of trust |  |  |
| Other |  |  |

19. What measures has the person who regularly sells you fish and/or seafood taken to avoid the likelihood of coronavirus transmission? (select all that apply).

- They wore a mask correctly (nose and mouth covered)
- They wore gloves
- Used hand sanitizer before handling the fish (even with gloves on)
- The person who delivers the fish and/or seafood is different from the person who collects the money
- None
- Other ________________________________________________

20. How do you regularly find out if a seller has the fish and/or seafood that you want to buy? (select all that apply in each column).

|  | BEFORE the lockdown | DURING the lockdown |
| --- | --- | --- |
|  | Answer | Answer |
| Whatsapp |  |  |
| Web app |  |  |
| Text message |  |  |
| Cell phone call |  |  |
| Webpage |  |  |
| Facebook |  |  |
| I ask my supplier directly in person |  |  |
| By notification from family or friends |  |  |
| None |  |  |
| Other |  |  |

21. How has the lockdown affected the prices of fish and/or seafood?

- They have decreased a lot
- They have decreased a little
- The prices are the same, they have not changed
- They have increased a little
- They have increased a lot
- Other ________________________________________________

22. Please select the option that best reflects your opinion, considering the period BEFORE the lockdown:

|  | Always | Sometimes | Never |
| --- | --- | --- | --- |
| Fish and/or seafood are available when I need them |  |  |  |
| The fish and/or seafood that I buy or consume is of good quality |  |  |  |
| The price of fish and/or seafood is affordable for my economy |  |  |  |

23. Please select the option that best reflects your opinion, considering the period DURING the lockdown:

|  | Always | Sometimes | Never |
| --- | --- | --- | --- |
| Fish and/or seafood are available when I need them |  |  |  |
| The fish and/or seafood that I buy or consume is of good quality |  |  |  |
| The price of fish and/or seafood is affordable for my economy |  |  |  |

24. Please select the option that best reflects your opinion, considering the period AFTER the lockdown (that is, once it has ended):

|  | Yes | Maybe | No |
| --- | --- | --- | --- |
| I will continue to consume fish and/or seafood at the same frequency |  |  |  |
| I will continue to buy fish and/or seafood from the same sellers |  |  |  |
| I will continue to use the same communication channels to find out if there are fish and/or seafood available |  |  |  |

25. Gender

- Male
- Female

26. Age group

- Under 18 years
- 18-25 years
- 26-35 years
- 36-45 years
- 46-60 years
- Over 60 years

27. What is your monthly income in USD?

- I do not receive any income at the moment
- Less than 500
- 501-1000
- 1001-1500
- 1501-2000
- 2001-2500
- 2501-3000
- 3001-3500
- 3501-4000
- 4001-5000
- More than 5000
- I prefer not to answer

28. Region of birth

- Sierra
- Coastal
- Amazon
- Insular (Galapagos)
- Foreign
- Other

# Supplementary Tables

**Table S1** General geographic and socioeconomic features of the three main coastal communities of the Galapagos Islands, Ecuador.

| Fishing port | **San Cristobal** | **Santa Cruz** | **Isabela** | **Total** |
| --- | --- | --- | --- | --- |
|  | Baquerizo Moreno | Puerto Ayora | Villamil | 3 |
| Population^1^ | 9667 | 20302 | 3071 | 33040 |
| Hotel capacity (beds)^2^ | 449 | 990 | 193 | 1632 |
| Restaurants and bars^2^ | 35 | 61 | 18 | 114 |
| Fishing license holders (active/registered)^3^ | 174/552 | 136/293 | 100/239 | 410/1084 |
| Fishing cooperatives | 2 | 2 | 1 | 4 |

^1^ INEC (2022)

^3^ Epler (2007)

^4^ Castrejón and Charles (2020)

**Table S2** Recategorization of ordinal variables (seafood consumption frequency and weekly average amount of seafood consumed) into numeric variables.

| **Variable** | **Category** | **Scale** |
| --- | --- | --- |
| Seafood consumption frequency | Never consume seafood | 1 |
|  | Once per month | 2 |
|  | Once every 15 days | 3 |
|  | Once per week | 4 |
|  | Twice per week | 5 |
|  | Three days per week | 6 |
|  | More than four days per week | 7 |
|  | Daily | 8 |
| Weekly average amount of seafood consumed | Half a pound or less | 1 |
|  | One pound | 2 |
|  | One pound and a half | 3 |
|  | Two to three pounds | 4 |
|  | More than three pounds | 5 |

**Table S3** Variable importance (VI) score (summing to 100) and ranking for each predictor variable of seafood frequency consumption of Galapagos residents, according to Boosted Regression Model. Deviance explained and the Pearson’s correlation coefficient are shown at the bottom of the table. Seafood: type of seafood consumed (fresh/frozen vs canned); EconSec: economic sector; HhDep: number of household dependents; Region: region of origin; Island: island of residence; MigrStat: migratory status; RN: random number.

| **Predictor variable** | **VI score** | **Ranking** |
| --- | --- | --- |
| Seafood | 27.6 | 1 |
| EconSec | 15.6 | 2 |
| Income | 15.3 | 3 |
| RN | 10.6 | 4 |
| HhDep | 8.7 | 5 |
| Age | 5.5 | 6 |
| Region | 5.4 | 7 |
| Island | 4.9 | 8 |
| Education | 2.1 | 9 |
| Period | 1.7 | 10 |
| Gender | 1.6 | 11 |
| MigrStat | 1.1 | 12 |
| Deviance explained (%) | 62.6 |  |
| Pearson’s correlation coefficient (r) | 0.60 |  |

**Table S4** Variable importance (VI) score (summing to 100) and ranking for each predictor variable of the amount of seafood consumed by Galapagos residents, according to Boosted Regression Model. Deviance explained and the Pearson’s correlation coefficient are shown at the bottom of the table. HhDep: number of household dependents; MigrStat: migratory status; RN: random number.

| **Predictor variable** | **VI score** | **Ranking** |
| --- | --- | --- |
| Sector | 24.6 | 1 |
| HhDep | 15.7 | 2 |
| RN | 13.5 | 3 |
| Income | 13 | 4 |
| Age | 10.6 | 5 |
| Region | 8.8 | 6 |
| Education | 6.3 | 7 |
| MigrStat | 2.7 | 8 |
| Gender | 2.1 | 9 |
| Period | 1.4 | 10 |
| Island | 1.4 | 11 |
| Deviance explained (%) | 56.22 |  |
| Pearson’s correlation coefficient (r) | 0.48 |  |

# Supplementary Figures


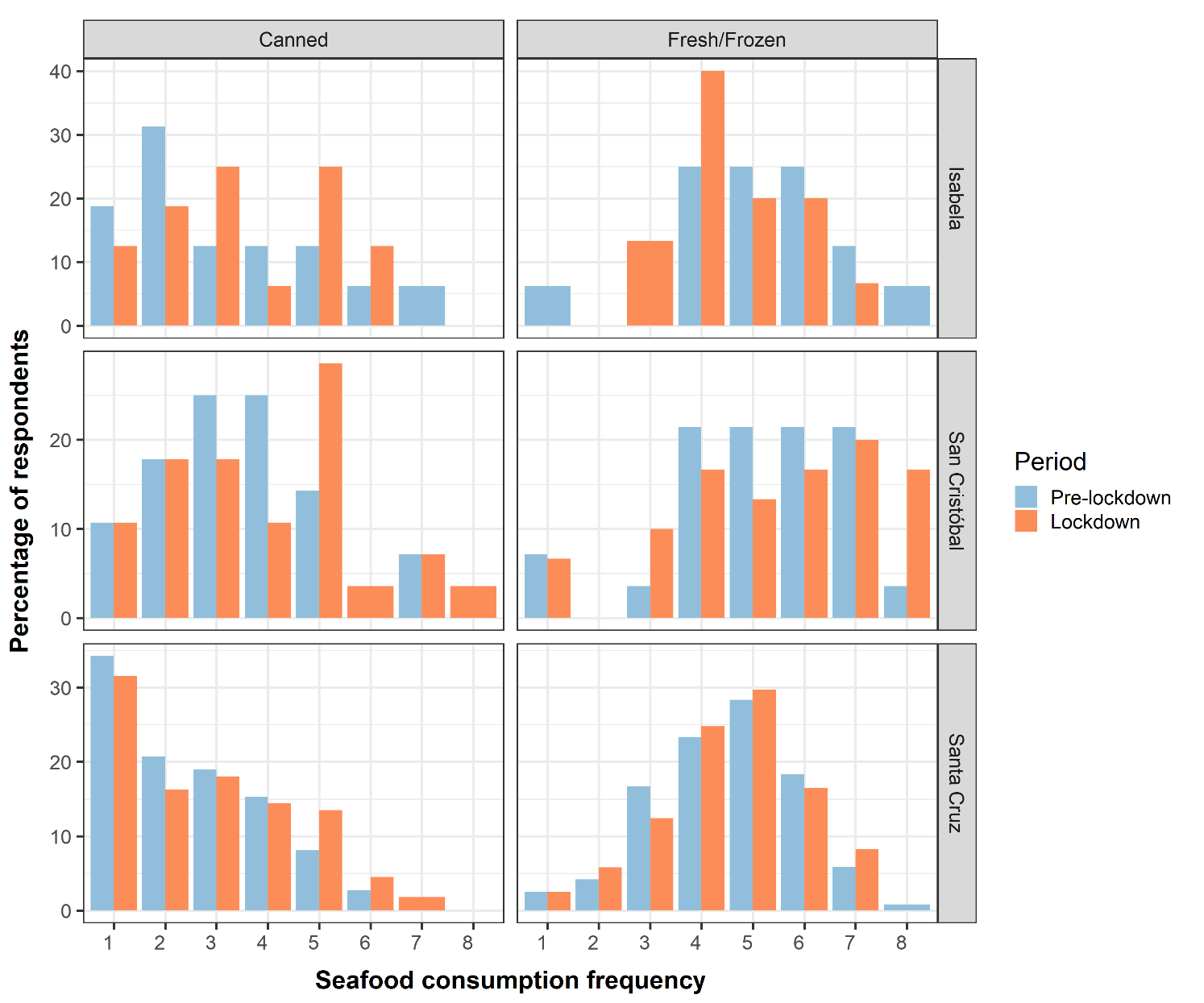


**Fig. S1** Seafood consumption frequency in the three main coastal communities of the Galapagos Islands, Ecuador, before and during the lockdown implemented from March 17th to July 1st, 2020. 1: never consume seafood; 2: once per month; 3: once every 15 days; 4: once per week; 5: twice per week; 6: three days per week; 7: more than four days per week; 8: daily.


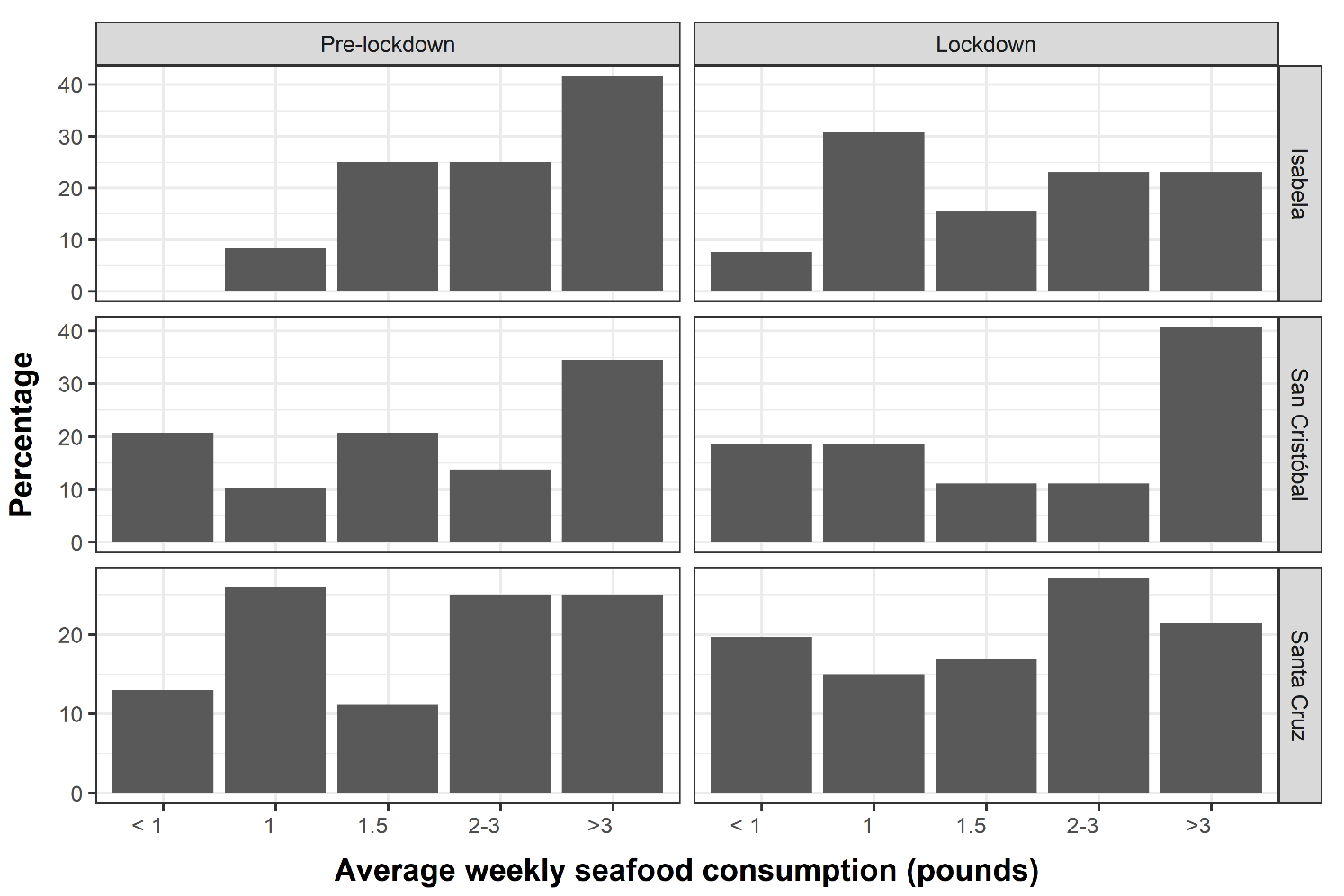


**Fig. S2** Average weekly seafood consumption (in pounds) in the three main coastal communities of the Galapagos Islands, Ecuador, before and during the lockdown implemented from March 17th to July 1st, 2020.


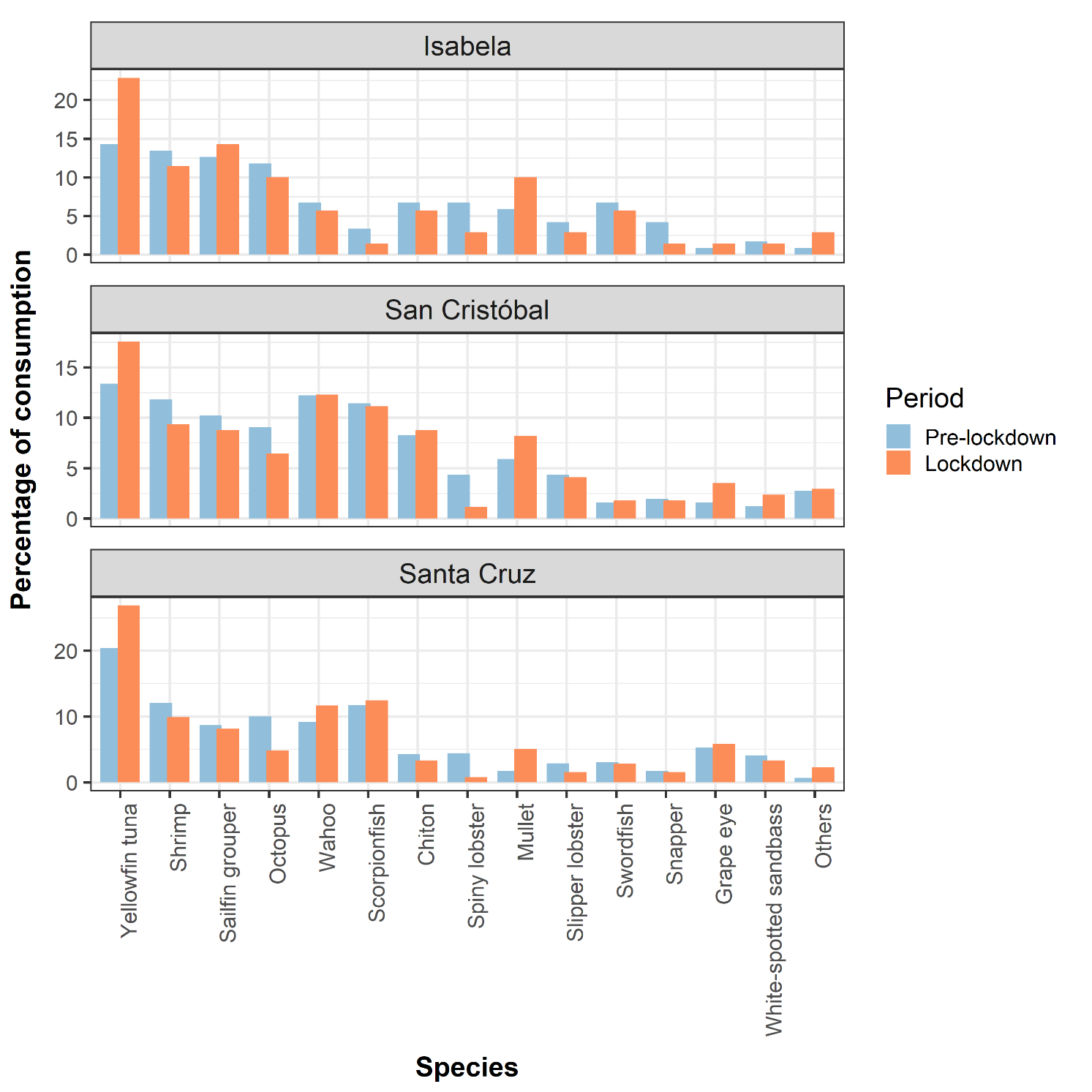


**Fig. S3** Variations in the percentage of aquatic species consumed in Isabela, San Cristobal, and Santa Cruz before and during the lockdown implemented in the Galapagos Islands from March 17th to July 1st, 2020.

# References

Castrejón, M., & Charles, A. (2020). Human and climatic drivers affect spatial fishing patterns in a multiple-use marine protected area: The Galapagos Marine Reserve. *PLoS ONE*, *15*(1), e0228094. https://doi.org/10.1371/journal.pone.0228094

Epler, B. (2007). *Tourism, economy, population growth, and conservation in Galapagos*. Charles Darwin Foundation.

INEC. (2022, September 16). *Proyección por edades provincias 2010-2020 y nacional*. Instituto Nacional de Estadísticas y Censos de Ecuador. https://www.ecuadorencifras.gob.ec/proyecciones-poblacionales/
